# NMR-derived secondary structure of the full-length *Ox40* mRNA 3′UTR and its multivalent binding to the immunoregulatory RBP Roquin

**DOI:** 10.1093/nar/gkac212

**Published:** 2022-03-31

**Authors:** Jan-Niklas Tants, Lea Marie Becker, François McNicoll, Michaela Müller-McNicoll, Andreas Schlundt

**Affiliations:** Goethe University Frankfurt, Institute for Molecular Biosciences and Biomagnetic Resonance Centre (BMRZ), Max-von-Laue-Str. 9, 60438 Frankfurt, Germany; Goethe University Frankfurt, Institute for Molecular Biosciences and Biomagnetic Resonance Centre (BMRZ), Max-von-Laue-Str. 9, 60438 Frankfurt, Germany; Goethe University Frankfurt, Institute for Molecular Biosciences, Max-von-Laue-Str. 13, 60438 Frankfurt, Germany; Goethe University Frankfurt, Institute for Molecular Biosciences, Max-von-Laue-Str. 13, 60438 Frankfurt, Germany; Goethe University Frankfurt, Institute for Molecular Biosciences and Biomagnetic Resonance Centre (BMRZ), Max-von-Laue-Str. 9, 60438 Frankfurt, Germany

## Abstract

Control of posttranscriptional mRNA decay is a crucial determinant of cell homeostasis and differentiation. mRNA lifetime is governed by *cis*-regulatory elements in their 3′ untranslated regions (UTR). Despite ongoing progress in the identification of *cis* elements we have little knowledge about the functional and structural integration of multiple elements in 3′UTR regulatory hubs and their recognition by mRNA-binding proteins (RBPs). Structural analyses are complicated by inconsistent mapping and prediction of RNA fold, by dynamics, and size. We here, for the first time, provide the secondary structure of a complete mRNA 3′UTR. We use NMR spectroscopy in a divide-and-conquer strategy complemented with SAXS, In-line probing and SHAPE-seq applied to the 3′UTR of *Ox40* mRNA, which encodes a T-cell co-receptor repressed by the protein Roquin. We provide contributions of RNA elements to Roquin-binding. The protein uses its extended bi-modal ROQ domain to sequentially engage in a 2:1 stoichiometry with a 3′UTR core motif. We observe differential binding of Roquin to decay elements depending on their structural embedment. Our data underpins the importance of studying RNA regulation in a full sequence and structural context. This study serves as a paradigm for an approach in analysing structured RNA-regulatory hubs and their binding by RBPs.

## INTRODUCTION

Cellular homeostasis strongly depends on the tight control of mRNA levels. Increased levels of specific mRNAs and, subsequently, their gene products have been found as a major cause for numerous diseases and types of cancer (1–4). The fate of a mature mRNA is regulated through *cis*-regulatory elements located in its 3′ untranslated region (UTR). 3′UTR *cis* elements mediate interactions with miRNAs and mRNA-binding proteins (RBPs) that govern mRNA fate, e.g. in tissue development and during immune response (5). One of the most abundant motifs are adenylate-uridylate-rich elements (ARE) (6); repetitive RNA stretches that exhibit a mainly destabilizing effect on mRNAs (7). 3′UTRs cover a large range of length (8), but there are only few studies that have addressed the functional context of an entire 3′UTR in detail (9).

The functional role of mRNA structure, specifically within 3′UTRs has become focus of research over the past years (8,10,11). The availability of *cis* elements governed by the RNA structural context affects translation and degradation (12), and it was shown that structured RNA elements in 3′UTRs enhance RNA maturation and polyadenylation (13). RNA regulation via structured *cis* elements involves the specific, shape-dependent recognition by cognate RNA-binding domains (RBDs) (14–17). The SLBP e.g. binds to 3′-located stem–loops of histone mRNAs with a very high affinity, a crucial event for histone pre-mRNA processing (18–20).

Despite ongoing progress in the identification of (structured) *cis* elements we have little knowledge about the structural and functional integration of multiple elements. Widespread 3′UTR structuring can lead to the clustering or occlusion of *cis* elements despite their distal localization of motifs (21–23). Their spatial arrangement accounts for the specific interaction with regulatory *trans*-acting RBPs. The majority of RBPs is of a multi-domain (MD) nature (24,25), which allows them to interact with 3′UTRs through multiple sites. The necessary structural analysis of such 3′UTR regulatory hubs has been hampered by their imprecise mapping, unreliable prediction of large RNA folds, by RNA dynamics, and their size. High-resolution structures of RNAs exceeding 100 nt are rare, e.g. the structures of a viral IRES of 107 nt (26) and the HIV-1 packaging signal of 151 nt (27). Low-resolution models, e.g. obtained from SAXS, can address RNAs of several hundred nt (28). Recently, viral RNA UTRs and lncRNAs have moved into the focus of research (28–30) while we are missing structural descriptions of full eukaryotic 3′UTRs.

Size and flexibility of RNAs often limit the use of conventional structural biology methods. Latest advances in Cryo-EM combined with chemical mapping and modelling circumvent these issues and provide high-resolution information (31). Su *et al.* could determine a 3.1 Å resolution structure of a 409-nt ribozyme solely based on Cryo-EM (32). At the same time wet-lab based approaches combining experimental restraints with bioinformatic predictions provide a suitable path to structural information (33) and similarly, ab initio *in silico* predictions have proven valuable for numerous RNAs (34).

**Figure 1.**
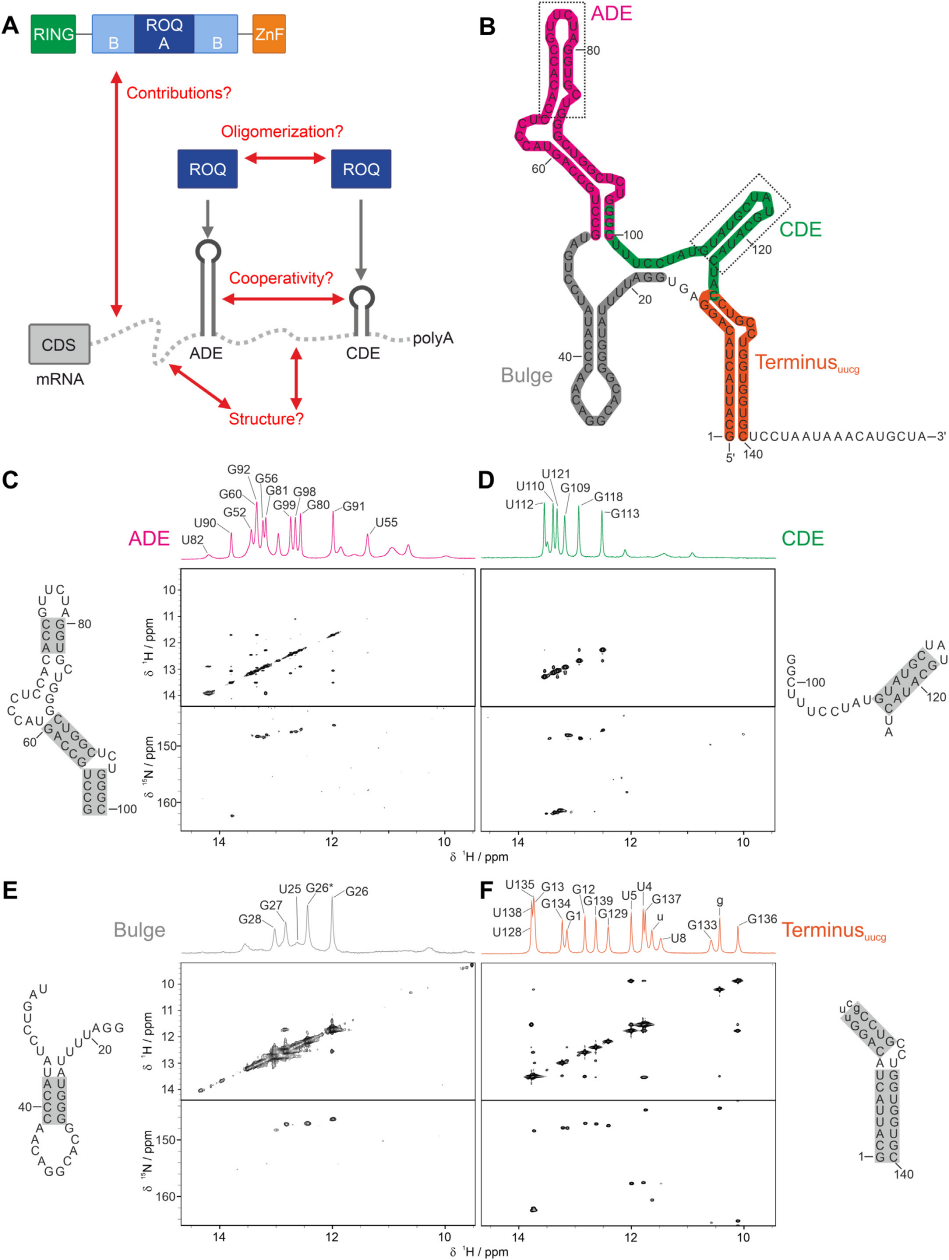
Structural characterization of *Ox40* 3′UTR fragments. (**A**) Potential network of interactions in a Roquin-*Ox40* 3′UTR complex. Both intra- as well as intermolecular interactions remain elusive. (**B**) Secondary structure prediction of the *Ox40* full-length 3′UTR. Elements used for structural analyses are coloured. Dashed boxes indicate elements described before (15). (**C**) ^1^H,^1^H-NOESY and ^1^H,^15^N-SOFAST-HMQC spectra of the ADE. Assignments of residues are shown in the ^1^H spectrum on top. Grey boxes in the secondary structure on the left indicate confirmed base pairs. The same experiments were recorded for the CDE (**D**), the Bulge (**E**) and the Terminus (**F**), which comprises an artificial UUCG-tetra-loop. Spectra are plotted within identical regions for comparison. The asterisk in (E) denotes a second conformation of G26. See also Supplementary Figure S1D.

The mRNA lifetime involves its controlled degradation by ribonucleases (35). Those are recruited by regulatory RBPs without an intrinsic nuclease activity, such as the proteins Roquin, TTP or Nanos (35,36). The MD-RBP Roquin, a CCCH-type zinc finger protein, is a repressive key regulator of the innate immune response (37–42), but has been found to affect mRNAs of other contexts as well and potentially controls cancer cell proliferation (43,44). Through its unique bi-functional ROQ domain (Figure 1A), it binds to 20–25 nt-sized stem–loops in target 3′UTRs (14). One such, the constitutive decay element (CDE), comprises a stemmed tri-loop structure (45), while Janowski et al. found a U-rich hexa-loop-containing stem–loop, which they termed alternative decay element (ADE) (15). There is increasing evidence that the core ROQ domain (‘A-site’) binds its target stem–loops by shape recognition, rather than sequence-dependent (43,46). Further Roquin target *cis* elements have been proposed recently (43,44,47–49) marking Roquin as a key regulator of mRNA stability.

Crystal structures suggested formation of a second, extended RNA-binding interface involving the N- and C-terminal HEPN-like regions of the ROQ domain (50), herein referred to as extended ROQ (extROQ), that provides an interaction platform for double-stranded RNA (dsRNA, ‘B-site’, Figure 1A). We currently have no experimental evidence for the simultaneous binding of Roquin to stem–looped and dsRNA in a natural 3′UTR context. We also lack a structural proof for the simultaneous binding of Roquin to more than one stem–loop in a target mRNA at a time, while multiple studies have described the interaction of Roquin with isolated ADE and CDE elements (14,15,47,50–52). In fact most target mRNAs of Roquin comprise 3′UTRs which integrate multiple decay elements arranged within varying distances and in different structural contexts, e.g. *ICOS, Ox40*, *Tnf*, *Nfκbiz* and *Nfκbid* (23,53).

The mRNA of the T-cell co-receptor *Ox40* (TNFRSF4 or CD134) comprises a highly conserved 3′UTR. *Ox40* is a negatively regulated target of Roquin and initiates immune responses through maintenance and differentiation of T-cells (40). The 3′UTR of 157 nt comprises an ADE and CDE, which both have been characterized in previous studies (15). In mouse embryonic fibroblasts either of the CDE or ADE sufficiently induced Roquin-mediated mRNA suppression with an additive effect from their combined presence. Ultimately, the positive and negative regulation of *Ox40* mRNA by various *trans*-acting factors (2) suggested the embedment of CDE and ADE elements within a larger structure context. The existence of additional *cis* RNA elements will then challenge the involvement of MD-RBPs like Roquin, and their possible oligomers (54,55). As a consequence, *Ox40* regulation will only be understood by unravelling its complex network of RNA-protein interactions based on a full description of the 3′UTR structure.

The precise combinatory arrangement and action of multiple decay elements in one 3′UTR towards mRNA lifetime remains elusive for literally all mRNAs. We here for the first time provide the secondary structure and geometric dimensions of an entire 3′UTR exemplified by the mRNA of *Ox40* (Figure 1B). Guided by NMR spectroscopy and supported by SAXS we show that the *Ox40* 3′UTR comprises widespread secondary structure that can be divided into four main structural elements. Elements fold independently from each other proven by individually trackable base pairs. We also probe the role of individual Roquin domains for the interaction with the 3′UTR elements. Based on a quantitative analysis, we suggest a mechanism of how Roquin uses its RBDs to engage with the *Ox40* mRNA 3′UTR in a functionally relevant sequence and stoichiometry. We show that an extended interface increases affinity through parallel stem binding, in which two Roquin molecules can bind the 3′UTR simultaneously. Finally, we give evidence for an affinity-enhancing function of an RNA element in 5′ of the CDE and determine a sequential binding mode of CDE and ADE.

## MATERIALS AND METHODS

### Cloning

All protein constructs used in this study have been cloned, expressed and initially analyzed before (14,56). RNA construct design was based on secondary structure predictions from mfold (57) and RNAfold (58). For both webservers, the folds of the ADE and CDE derived from Janowski et al. were used as folding restraints, respectively (15). Folding was performed at 37°C and a window parameter of 10 was used for mfold. All other parameters for both mfold and RNAfold were left as default. Templates for RNA *in vitro* transcription were generated via Gibson restriction free cloning of PCR products with compatible overhangs into the sPHDV64 vector containing the T7 promotor sequence and the HDV ribozyme at the 3′ end. Briefly, PCR products obtained from the full-length mouse *Ox40* mRNA 3′UTR (NCBI entry XM_006538724.4) vector with different primers were incubated with PCR-amplified linear vector at 50°C for 60 min in presence of T5 exonuclease, Phusion polymerase and Tag DNA Ligase and subsequently transformed into chemically competent DH5α cells. Plasmids were isolated via Miniprep according to the vendor's protocol (Promega) from a 100 μg/ml Ampicillin overnight culture and constructs were confirmed by sequencing. The full-length 3′UTR comprises wildtype residues 1078–1234 which are labelled 1–157 for convenience in this manuscript. All other fragments and their corresponding residues are listed in Supplementary Table S1.

### Protein expression and purification

For protein expression of murine Roquin-1 constructs a single, freshly transformed colony of *Escherichia coli* BL21 (DE3) cells was used to inoculate a 50 ml LB pre-culture with 50 μg/ml Kanamycine and incubated by overnight shaking at 37°C. The complete volume was transferred to 1 L LB with Kanamycine, and protein expression was induced with 1 mM IPTG at an OD_600_ of 0.7–0.9. Proteins were expressed overnight at 37°C and harvested by centrifugation at 4°C and 3000 g for 25 min. For ^15^N labelling 2 ml of the pre-culture were used to inoculate one liter of M9 minimal medium containing 1 g/l ^15^N NH_4_Cl with Kanamycine. Proteins containing the RING or ZnF domain were expressed in presence of 100 μM ZnCl_2_ (Supplementary Table S2). Cells were resuspended in lysis buffer (50 mM Tris pH 8.0, 500 mM NaCl, 4 mM β-mercaptoethanol, 0.05% sodium azide) supplemented with 3 mg Protease Inhibitor Mix G (SERVA) and DNase I, and lysed using sonification for ten times 1 min with an amplitude of 70% and 0.8 s pulses at 4°C. Buffers for purification of RING or ZnF containing proteins were supplemented with 50 μM ZnCl_2_. After centrifugation at 12 500 g for 30 min at 4°C the supernatant was applied to a Ni-NTA column. The column was washed with 2–3 column volumes of lysis buffer with increasing amounts of imidazole (50, 100, 300, 500 mM) and protein elution was monitored via SDS-PAGE. The protein was cleaved using 25 nmol in-house made TEV protease per liter of original culture while dialysis against 50 mM Tris pH 8.0, 1 M NaCl overnight. The cleaved protein was purified from tags using a reverse Ni-NTA column in dialysis buffer with increasing imidazole concentrations. The protein fraction was concentrated using Amicon® centrifugal filter units and finally subjected to SEC (HiLoad S75 16/600 or S650 16/600 column from GE depending on the molecular weight) run in 1 M NaCl, 20 mM Tris pH 7.0, 2 mM TCEP. Purity was confirmed by SDS-PAGE (Supplementary Figure S4A) and the isolated proteins were frozen in liquid nitrogen and stored at –80°C until usage.

### *In vitro* transcription and RNA purification

Templates for *in vitro* transcription were obtained from Gibson restriction-free cloning of PCR products into the sPHDV64 vector. RNAs were transcribed from plasmids isolated from *E. coli* DH5α cells using Qiafilter Plasmid GigaKits (Qiagen) of 3 l cultures grown overnight and supplemented with 100 μg/ml Ampicillin following the manufacturer's instructions. Template plasmids were linearized prior to in vitro transcription. Template DNA of 0.1 μg/μl was mixed with 4 mM of each NTP, 50 mM Tris–HCl pH 7.5, 40 mM MgCl_2_, 5 mM DTT, 2 mM Spermidine, 400 nM in-house made T7 RNA polymerase and ddH_2_O. The mixture was incubated at 37°C for 3–4 h and after addition of EDTA, in order to to dissolve Mg^2+^ salts, RNA was precipitated at -20°C for two hours by adding 0.1× volume 3 M sodium acetate pH 7.5 and 1× volume isopropanol. The RNA was centrifuged at 4°C and 14 000 g for 30 min, the supernatant was discarded and the pellet air-dried. The RNA was resuspended in 2 ml ddH_2_O, mixed with 1 ml of loading buffer (8 M urea, 1 mM EDTA pH 8.0, 30% glycerol) and loaded onto a 10–20% acrylamide urea gel (8 M urea, 1× TBE, 19:1 acrylamide:bis-acrylamide) depending on the molecular weight of the RNA. Gels were run for 14–16 h at 300 V in 1× TBE buffer. The bands of interest were cut under UV shadowing and the RNA was eluted from gel slices by crush and soak in 0.3 M sodium acetate pH 7.5 oN at room temperature. RNAs were eluted twice and the supernatant collected, passed through a 0.45 μM filter, concentrated and washed ten times with ddH_2_O in an Amicon^®^ centrifugal filter unit of adequate molecular weight cut off. The RNA was then dried in a lyophilizer, dissolved in water or buffer and stored at –80°C after snap cooling.

### Native RNA purification

For native purification IVT templates were generated by PCR from plasmids mentioned above using primers to omit the HDV sequence (forward primer with reverse full-length or reverse CDE, Supplementary Table S3). After *in vitro* transcription the mixture was centrifuged at 14 000 g and 4°C for 30 min to pellet precipitated Mg complexes. The supernatant was carefully decanted and passed through a 0.45 μm filter unit. Samples were purified via SEC using S650 16/600 (GE Healthcare, for full-length) and Enrich S70 10/300 (BioRad, for Bulge-ADE–CDE) columns and concentrated in an Amicon® centrifugal filter unit for NMR measurements.

### CD spectroscopy

CD spectra of RNAs were recorded in a JASCO J-810 CD spectrometer using 10 – 30 μM samples in 150 mM NaCl, 20 mM Tris pH 7.0, 2 mM TCEP in High Precision Cell Quartz cuvettes with a 1 mm path (Hellma Analytics) at wavelengths from 230 to 300 nm (Data not shown), with a data interval of 0.5 nm. Melting curves were recorded from 5 to 95°C at 260 nm in 0.1°C intervals with a temperature ramp of 1 degree / min. All data was normalized to 5°C. Data was plotted and melting points were calculated with Origin using a bi-dose response curve fit to cover the full temperature range. For selected RNAs the first derivative of the CD signal was calculated and plotted.

### NMR spectroscopy

Proteins were buffer exchanged to 150 mM NaCl, 20 mM Tris pH 7.0, 2 mM TCEP using an Amicon® centrifugal filter unit prior to measurements. ^1^H,^15^N-HSQC spectra of 50 μM ROQ alone and in presence of 0.5, 1.0 and 1.3-fold amounts of ADE, CDE or tandem ADE–CDE RNA were recorded at 293 K on a 600 MHz spectrometer (Bruker). Lyophilized RNAs were dissolved in 50 mM KCl, 25 mM sodium phosphate pH 7.0 and refolded by snap cooling. 1D proton spectra and all experiments on protein-RNA complexes were recorded at 293 K. ^1^H,^1^H-NOESY, ^15^N-SOFAST-HMQC (59) and further 1D proton experiments for assignments were recorded at 283 K to stabilize structures. RNA concentrations for 2D spectra ranged from 300 to 600 μM, while for 1D experiments concentrations as low as 20 μM were used. All experiments were recorded on Bruker AV spectrometers with 600, 700, 800, 900 and 950 MHz proton Larmor frequencies equipped with triple-resonance cryoprobes. For ^1^H,^1^H-NOESY spectra the mixing times were set to 150 or 250 msec depending on the size of the RNA, and between 320 and 600 indirect points were used for acquisition. ^1^H,^15^N-SOFAST-HMQC spectra were recorded to unambiguously distinguish Uracils from Guanines. All spectra were processed using Topspin 4.0.6. (Bruker) and analyzed with NMRFAM-Sparky 1.414 (60).

### In-line probing

For in-line probing fluorescently labelled RNA was used. *Ox40* full-length 3′UTR was dephosphorylated with calf intestinal phosphatase at 50°C for 30 min. After phenol/chloroform extraction and ethanol precipitation the RNA was 5′ end-labelled with ATP-γ-S and T4 polynucleotide kinase (PNK) overnight at 37°C according to (61). After a second phenol/chloroform extraction and ethanol precipitation the RNA was resuspended in 25 mM HEPES pH 7.4 and labelled with 7.5 mM 5-Iodoacetamidofluorescein (IAF) dissolved in DMSO. The mixture was incubated for 4 h in the dark. The labelled RNA was purified from a denaturing polyacrylamide gel (see above). After elution the RNA was washed with water in an Amicon^®^ centrifugal filter unit and concentrated. In-line probing was performed as described before (62). Briefly, for in-line probing 60 pmol of refolded RNA were incubated in 50 mM Tris–HCl pH 8.3, 20 mM MgCl_2_ and 100 mM KCl at room temperature for 24 h in the dark either alone or in presence of 180 pmol coreROQ or extROQ. A G-ladder was generated by incubating 60 pmol of RNA with of 0.1 U T1 RNAse in 25 mM sodium citrate pH 5.0 for 6 min at 55°C. For the alkalic ladder 60 pmol of RNA were incubated with 50 mM Na_2_CO_3_ pH 9.0 and 1 mM EDTA for 6 min at 90°C. All samples were mixed with loading buffer (see above) and stored on ice to stop the reaction. The samples were loaded onto a 10% polyacrylamide gel (19:1) with 8 M urea which was run for 3 h at constant 40 W. Gels were scanned with a Typhoon 9400 Variable Mode Image (GE).

### *In vitro* SHAPE-seq

A modified full-length *Ox40* 3′UTR RNA (178 nt) was produced as described above, containing a 3′ adapter for reverse transcription (5′-GATCGGAAGAGCACACGTCTG-3′, Supplementary Table S5). Structural integrity was confirmed in an imino-proton 1D NMR spectrum (Supplementary Figure S2A). For the SHAPE (Selective 2′-hydroxyl acylation analyzed by primer extension) reaction, 3 pmol of the RNA was refolded in 27 μl of 50 mM KCl, 25 mM NaPi, pH 8.0. Three μl of 1-methyl-7-nitroisatoic anhydride (1M7) or *N*-methylisatoic anhydride (NMIA) in DMSO was added to the sample to a final concentration of 10 mM (63). Two replicates were prepared for each condition. Samples were incubated at 37°C for 2 min for 1M7 and 40 min for NMIA and afterwards stored on ice. RNAs were concentrated by precipitation, dissolved in 10 μl of distilled H_2_O and reverse transcribed using Superscript III (Invitrogen) and an RT primer complementary to the 3′ adapter. 10 μl diluted cDNA (DMSO 1:160, NMIA 1:80, 1M7 1:40) were used for PCR (5 cycles) in a 100-μl reaction using Phusion® High-Fidelity PCR Master Mix (New England Biolabs M0531S) and 0.5 μM forward and reverse primers (Supplementary Tables S6 and S7) adding Illumina adapters, experimental barcodes and random barcodes. PCR libraries were purified using the ProNex® Size-Selective Purification System (Promega NG2001) removing primers and cDNAs, and sequenced on an Illumina NovaSeq 6000 instrument (150-bp paired end reads; 15 million reads per sample; Novogene, Cambridge).

Reads were cleaned from adapters and barcodes and then mapped to the murine *Ox40* 3′UTR sequence using the STAR aligner (64) preventing soft-clipping and mismatches. 15–20% of the reads could not be aligned because they contained mismatches. These unmapped reads were kept and remapped with STAR, this time allowing up to 10 mismatches. The numbers of substitutions (base changes) and deletions/insertions per nucleotide position were extracted from these alignments using the integrated genome viewer (IGV) and plotted normalized to the total number of reads. The most terminal 5′ends were not analyzed due to low data quality. Data was normalized to G13.

### EMSA

For electro mobility shift assays (EMSA) RNAs were dephosphorylated with calf intestinal phosphatase at 50°C for 30 min. After phenol/chloroform extraction and ethanol precipitation RNAs were radioactively phosphorylated at the 5′ end using [γ-^32^P]-ATP (Hartmann Analytics) and T4 polynucleotide kinase (PNK). After heat inactivation of T4 PNK RNAs were purified from excess γ-ATP using NucAway Spin columns (Invitrogen) according to the manufacturer's protocol and diluted using NMR buffer (150 mM NaCl, 20 mM Tris pH 7.0, 2 mM TCEP). After snap cooling RNAs were stored at –20°C. 2 μl of RNA were mixed with increasing amounts of protein (0, 20, 50, 100, 150, 200, 250, 300, 400, 500, 600, 700, 1000, 2000, 5000 or 0, 50, 100, 200, 300, 400, 500, 600, 700, 1000, 1500, 2000, 5000, 10 000, 20 000 nM), 0.6 μg tRNA_Phe_ from baker's yeast (Roche), 1 mM MgCl_2_ and buffer to yield 20 μl. After incubation for 15 min 3 μl of loading buffer (1 × TB, 60% glycerol, 0.02% bromphenolblue) were added. 10 μl of each sample were loaded on 6–8% acrylamide gels (37.5:1 acrylamide:bis-acrylamide, 5% glycerol, 1 × TB buffer) and gels were run at 80 V for 80–100 min in pre-chilled 1× TB buffer. Gels were dried for 2 h at 80°C in a Gel Dryer 543 (Biorad) and afterwards exposed to a phosphor storage screen (Amersham Biosciences) for 1–2 h. A Typhoon 9400 Variable Mode Image (GE) was used for detection and imaging of screens. Quantitative analysis was performed with ImageJ: noise-corrected intensities of the unbound RNA were subtracted from total RNA per lane to calculate the ratio of the bound fraction over total RNA according to the formula

$$\begin{equation*}{f_{bound}} = \ 1 - \ \frac{{{{{{I_{free}} - \ {N_{free}}}} \!\mathord{\left/ {\vphantom {{{I_{free}} - \ {N_{free}}} {{I_{total}} - \ {N_{total}}}}}\right.} \!{{{I_{total}} - \ {N_{total}}}}}}}{{{{{I{0_{free}} - \ {N_{free}}}} \!\mathord{\left/ {\vphantom {{I{0_{free}} - \ {N_{free}}} {I{0_{total}} - \ {N_{total}}}}}\right.} \!{{I{0_{total}} - \ {N_{total}}}}}}}\end{equation*}$$

where *I*_free_ and *I*_total_ are the intensities of the free and total RNA per lane, respectively, and *N* is the noise. I0 denotes the intensity at zero protein concentration for normalization. Data was plotted and *K*_d_ values calculated using the software Origin.

### Small angle X-ray scattering (SAXS)

Proteins were buffer exchanged to Roquin NMR buffer (150 mM NaCl, 20 mM Tris pH 7.0, 2 mM TCEP) using an Amicon^®^ filter unit. Samples of 50 μl were frozen in liquid nitrogen for transport on dry ice. Measurements were carried out at beamline P12 (PETRA III) of DESY, Hamburg (65). The flow-through collected from the filter units was used as buffer reference. Similarly, RNAs were buffer exchanged to RNA NMR buffer (50 mM KCl, 25 mM NaPO_4_ pH 7.0). Measurements were undertaken at 293 K. For data processing the ATSAS (66) package was used. *D*_max_, *R*_g_ and Porod volumes were determined using the Primus tool and derived from the molecular weight wizard. For molecular weight estimation we employed the implemented Bayesian approach (67). Structural models were generated with *RNAMasonry* (68) using NMR-derived base pairs as restraints and optimized for fit with the experimentally obtained SAXS curves. For each RNA 50 simulation steps were performed. The given, final *X*^2^ values were calculated with the ATSAS-implemented CRYSOL (69) tool.

## RESULTS

### The *Ox40* mRNA 3′UTR reveals an extended secondary structure

For the first structural assessment of a full-length 3′UTR and its interactions with a regulatory RBP we chose the mRNA of the crucial T-cell co-receptor *Ox40*, a known target of the suppressive immune-regulator Roquin (40). We first set out to determine all potential RNA *cis*-regulatory elements within the full-length *Ox40* 3′UTR. The 3′UTR has a size of 157 nt that renders it addressable by structural studies and suggests it a paradigm as autarchic regulatory hub, which would potentially be present in long 3′UTRs of other mRNAs. Secondary structure predictions using the programs *mfold* (57) and *Vienna RNAfold* (58) unequivocally suggested four structured main elements (Supplementary Figure S1A): A 5′ located element, which we termed Bulge, the ADE, the CDE and the kinked-helical Terminus preceding an unpaired stretch of 17 nt. The ADE here is in fact an extended helix-stem–loop fold which includes the recently introduced ADE hexa-loop (15) at its apical end (here termed ADE_short_, Supplementary Figure S1B). The ADE is predicted to be highly stable, yet with a flexible region within the central stem indicated by low scores in *Vienna RNAfold*. In contrast, predicted folds for both Bulge and CDE are less convergent. The Terminus predicted to form a bulged duplex while the flexible tail potentially allows to interfere with nearby secondary structure. Given those ambiguities, we designed a set of *Ox40* 3′UTR constructs for subsequent secondary structure determination based on the energetically most prominent predicted fold (Table 1, Figure 1B, Supplementary Figure S1B). Our constructs thereby covered both small isolated elements and logical combinations of them.

**Table 1.** SAXS-derived parameters and melting points obtained from CD spectroscopy of *Ox40* 3′UTR RNA constructs. Theoretical molecular weights were obtained from (97). n.d. = not determined

RNA	*T*_m_ (°C)	Theoretical MW (kDa)	MW Bayesian (kDa)	*R*_g_ (nm)	Porod vol. (Å^3^)	*D*_max_ (nm) from SAXS	*D*_max_ (nm) from model
**ADE**	74.7 ± 0.1	15.84	16.13	2.12	20 925	6.24	6.95
**ADE_short_**	n.d.	7.23	7.30	1.67	8 766	5.38	4.36^a^
**CDE**	68.6 ± 0.1	9.36	11.25	2.05	12 947	10.57	6.65
**CDE_short_**	n.d.	4.96	7.30	1.48	7 042	4.42	3.90
**ADE–CDE**	72.9 ± 0.1	24.05	19.93	2.64	25 759	9.60	9.22
**Bulge**	52.2 ± 0.6	11.43	11.25	1.83	12 980	5.54	6.41
**Bulge-ADE**	n.d.	27.11	25.57	3.27	34 261	12.29	11.67
**Bulge-ADE–CDE**	n.d.	35.32	31.65	3.36	39 669	11.80	10.89
**Terminus_UUCG_**	58.6 ± 0.1	10.43	10.40	1.63	13 998	5.47	5.55
**Full-Length**	56.9 ± 0.8	50.40	47.68	3.73	57 539	12.48	11.65
	73.3 ± 0.3			(3.91)^b^			

^a^ Taken from ADE in (15).

^b^ Value in parentheses derived from *RNAMasonry*-built model.

All RNAs were produced by *in vitro* transcription (IVT), purified by denaturing PAGE (Supplementary Figure S1C) and refolded via snap-cooling. To exclude the potential formation of RNA oligomers during refolding we conducted SAXS experiments with all RNAs. The SAXS-derivable molecular weights confirmed their monomeric states, respectively (Table 1). We next used solution NMR spectroscopy to monitor secondary structure formation of isolated RNA elements based on the detectability of individual imino protons, indicative of base-pairing (Figure 1C–F). We found an excellent spectral quality allowing to assign the majority of imino protons and nitrogens. To this end, we recorded ^1^H,^1^H-NOESY (yielding connectivities between neighbouring iminos) and ^1^H,^15^N-SOFAST-HMQC spectra (unambiguously revealing Gs and Us). Overall NMR line-widths are in agreement with monomeric RNAs and allowed the near-complete assignment of individual imino protons (exemplified by the CDE in Supplementary Figure S1D). For all four elements (ADE, CDE, Bulge and Terminus), the assignments suggested a secondary structure along with the prediction shown in Figure 1B. Importantly, 1D proton spectra of the short ADE and CDE fully represent the signal patterns obtained with synthetic RNAs in earlier work (15).

For the ADE the lack of cross peaks in the NOESY spectrum indicates that residues 61–70 and 83–88 are not part of a stable duplex or a single secondary structure. This is in agreement with the ambiguous predictions in Supplementary Figure S1A. However, the presence of resonances at 10–12 ppm (Figure 1C) caused by GU base pairs suggests a transient double-stranded secondary structure in line with the fold shown in Figure 1B.

All CDE imino proton resonances could be assigned unambiguously and point at a single stable conformation and a fold in agreement with the prediction. However, we found additional weak signals for the CDE (Figure 1D) that are likely caused by the extended unstructured termini, which had not been part of previous studies (15).

We found few, but sharp lines for the Bulge in the 1D proton spectrum (Figure 1E) underlining its general presence as a folded element within the *Ox40* 3′UTR. We were able to assign three GC base pairs and the preceding A42-U25. A second conformation indicates the dynamics within the possible AU-stretch. Consequently, G26-C41 appears with a second imino group correlation (with an open preceding AU pair), while we found additional, broadened peaks in the proton spectra. We therefore suggest the bulge is a rather flexible and dynamic region with respect to the NMR time scale but could still serve as a specific element to be bound and stabilized by a cognate RBD as was shown for AU-rich folded RNAs before (47).

For the Terminus, we designed a construct to be used in a convenient one-step IVT, enabled by a UUCG-tetraloop at its artificial apical end (Figure 1B, Supplementary Figure S1B). This strategy has been applied before (29,70) in order to minimize the possible conformational space for the RNA when excised from its full-length, more restrictive context. Indeed, we successfully assigned the elongated stem harbouring a small bulge (Figure 1F) in a unique conformation.

### Thermal stabilities of *Ox40* 3′UTR RNA elements

**Figure 2.**
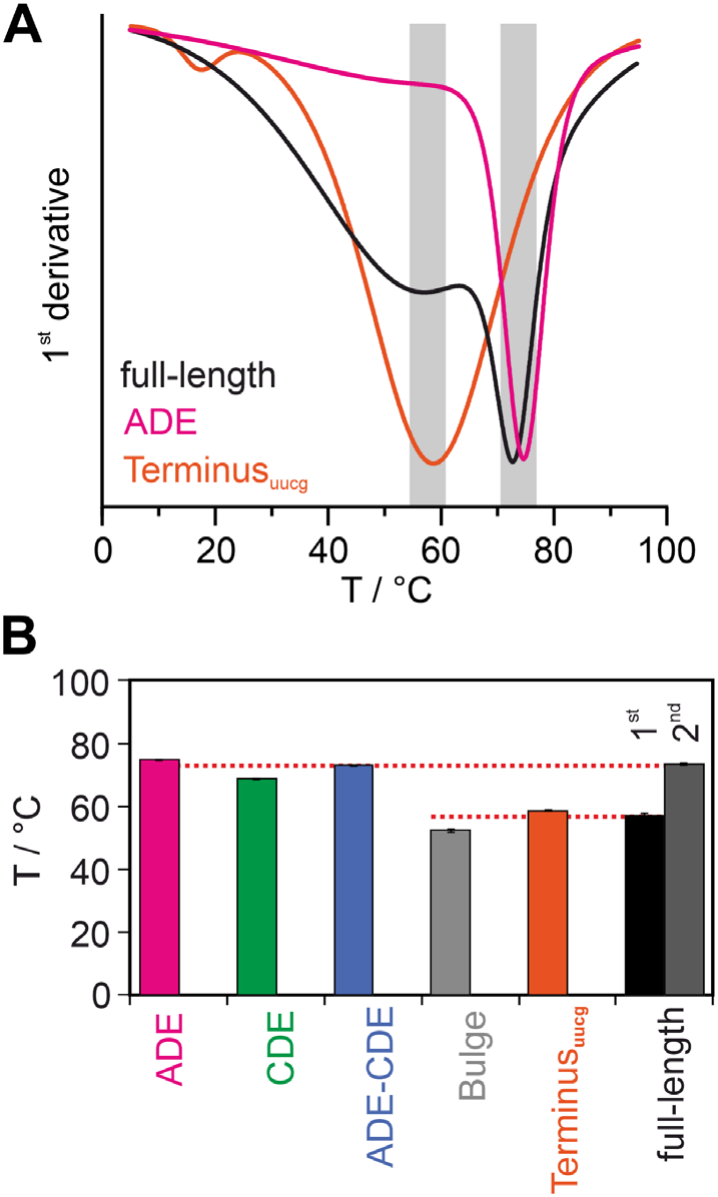
Thermal stability of RNA elements within the *Ox40* 3′UTR. (**A**) shows the first derivative of melting curves for three selected RNAs. Grey boxes indicate transition areas for the two prominent melting points (see Supplementary Figure S1 for details). (**B**) Bar plot of melting temperatures for all *Ox40* 3′UTR RNA elements as depicted.

To quantify stabilities of and confirm the suggested folds, we next measured temperature melting points (T_m_) of RNA elements using CD spectroscopy (Table 1, Figure 2A and B, Supplementary Figure S1E). In line with the data from imino NMR spectra, ADE and CDE were found as thermally stable elements with late T_m_ values in a similar range as for related decay elements, e.g. the CDE2 element in the 3′UTR of *UCP3* mRNA (47). In direct comparison, Bulge and Terminus are thermally less stable structures underlined by early *T*_m_ values. The Terminus, however, shows an excellent data fit which suggests a unique conformation. In contrast, the broad transition point and constant decrease of the Bulge CD signal confirms its labile structure and multiple conformations as also suggested by NMR.

Altogether, our data clearly support the presence of a compactly folded *Ox40* mRNA 3′UTR. Based on our data, we claim four dividable *Ox40* 3′UTR RNA elements (Figure 1B), of which the ADE and CDE represent stably folded moieties while the flanking Bulge provides a dynamic, transiently folded element. Our data suggest that the overall *Ox40* 3′UTR structure is rigidified and constraint by long-range RNA-RNA duplex interactions within the Terminus.

### The embedment of RNA elements within the full-length *Ox40* 3′UTR

**Figure 3.**
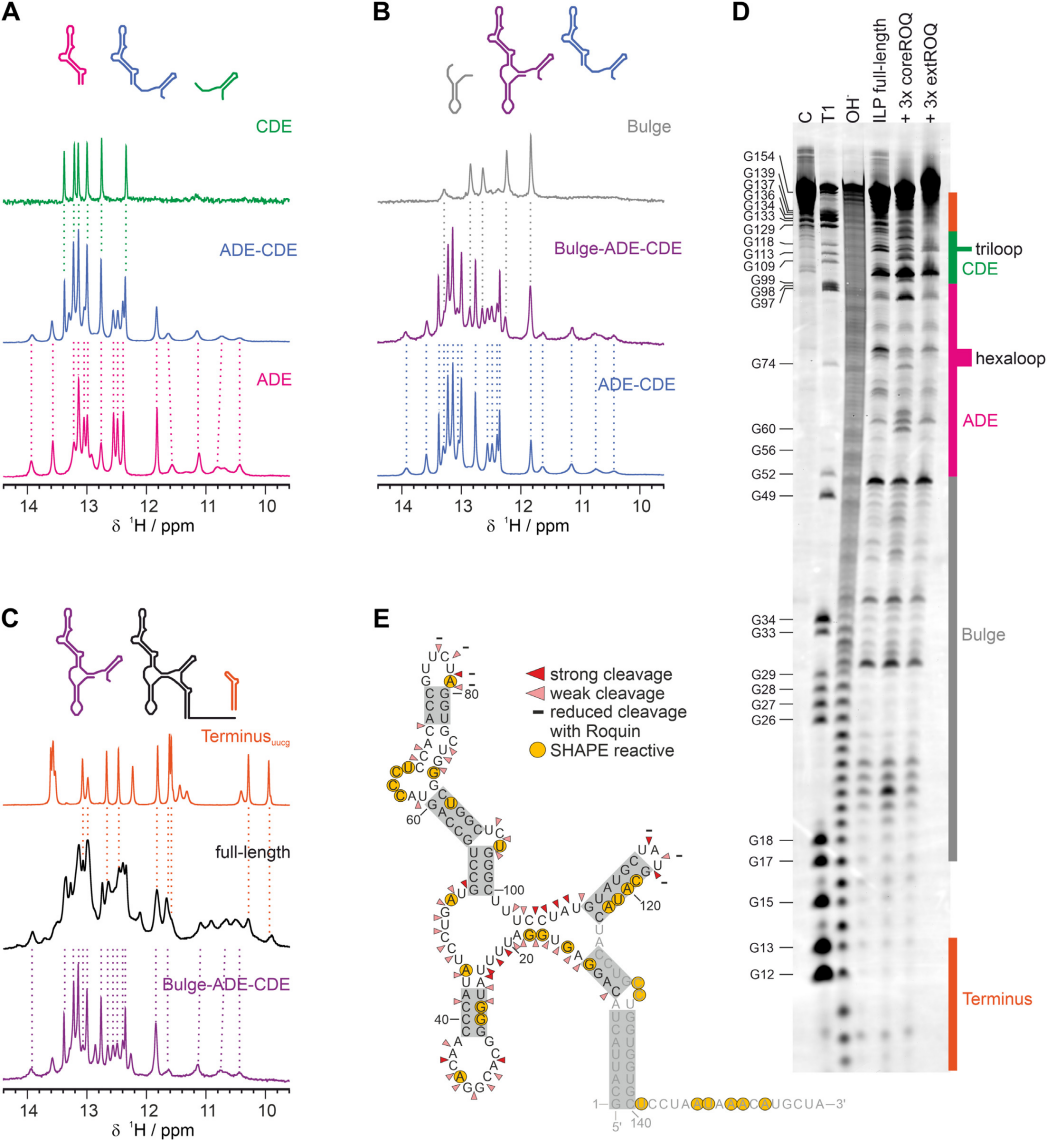
Modular architecture of the *Ox40* 3′UTR. (**A**) ^1^H spectra of CDE, tandem ADE–CDE and ADE. Dashed lines connect resonances of the tandem ADE–CDE spectrum with the corresponding ones in the CDE- and ADE-only spectra. The respective elements are depicted as cartoons above. The same comparison as in (A) is shown in (**B**) for the Bulge, Bulge-ADE–CDE and ADE–CDE construct and in (**C**) for the Terminus, full-length and the Bulge-ADE–CDE. (**D**) In-line probing of full-length *Ox40* 3′UTR. The gel shows an untreated control C, the T1-cleaved G-ladder, an alkaline ladder and the In-line probing reaction of the free RNA alone and in presence of coreROQ or extROQ (left to right). The guanine bases are labelled on the left; the corresponding RNA elements are colour-coded on the right. (**E**) Cleavage pattern of (D) mapped onto the secondary structure model of the *Ox40* 3′UTR. Triangles indicate weak and strong cleavage events. Grey boxes highlight base-pairs confirmed by NMR (compare to Figure 1). For residues shaded light grey no ILP information was obtained. Yellow circles display residues with SHAPE reactivity in both 1M7 and NMIA probing (see Supplementary Figure S2 for details).

We next asked for potential higher order structure of the four main elements in a full 3′UTR context. RNA-RNA interactions between elements will be unambiguously traceable and locatable by chemical shift changes of the imino proton resonances. We thus designed multi-element constructs and compared their 1D NMR spectra with those recorded from individual elements (Figure 3A–C, Supplementary Figure S1B and C, Supplementary Figure S3A and B). Judged by chemical shifts, 1D proton and 2D correlation spectra of individual elements remarkably overlapped with those recorded from extended constructs. Similarly, inspection of line-widths indicates that ADE, CDE, Bulge and Terminus in temporal average behave as independently folded elements. E.g., in fusion with other elements, the narrow lines of the CDE were retained (Figure 3A and B, Supplementary Figure S3A and B), whereas the Bulge-derived resonances were found line broadened independent of their context (Figure 3B and C). This suggests that the CDE tumbles independently and the Bulge remains flexible in the presence of other elements. Strikingly, sharp lines in the tandem ADE–CDE construct confirm the structural independence and robustness between these crucial two RNA decay elements (Figure 3A, Supplementary Figure S3A), which has been a matter of debate before (15). In particular, for the CDE independence is strongly supported by the NOESY spectrum of the full-length 3′UTR (Supplementary Figure S3B), which appears dominated by CDE-derived signals. We assume this is caused by narrow lines of CDE iminos relative to the average, broad line-width of the 3′UTR based on the molecular weight of the full-length RNA. This finding is in line with an overlay of ^1^H,^15^N 2D NMR spectra detecting imino group correlations (Supplementary Figure S3A).

In line with NMR data, the conserved melting temperatures of elements in extended contexts expresses their structural independence. We observe only one transition in the melting curve of the tandem ADE–CDE RNA which is likely caused by a comparable *T*_m_ of the two isolated elements (Table 1, Figure 2B, Supplementary Figure S1E). Interestingly, for the full-length 3′UTR we clearly see two transitions corresponding to the early and late melting events at 56.9°C and 73.3°C. We conclude, the four elements appear grouped into pairs, with the Bulge and Terminus melting at the lower and ADE and CDE melting at the higher temperature, respectively. This thermal robustness is in line with a *Multiz* alignment of *Ox40* 3′UTRs across vertebrates revealing the high degree of conservation of both ADE and CDE (Supplementary Figure S6A and B).

We complemented our NMR experiments with In-line probing testing for single-stranded regions in the full-length 3′UTR context. The loops, Bulge and the central hinge region show cleavage bands indicative of single-stranded bases (Figure 3D and E). We could not observe significant cleavage in regions previously identified as double-stranded in our NMR experiments. Hence, In-Line Probing nicely confirms our NMR data of isolated RNA elements and combinations thereof. To obtain more quantitative information on accessible, i.e. potential single-stranded, regions we used SHAPE combined with deep sequencing (SHAPE-seq (71)) of the full-length 3′UTR (Figure 3E, Supplementary Figure S2). Probing the RNA with two distinct SHAPE-reagents—1M7 and NMIA—showed increased reactivity in the central region of the 3′UTR as well as in ADE bulges, loops and the tail. These results are in good agreement with our In-Line Probing data. Interestingly, the 3′ half of the CDE stem shows elevated reactivity. We speculate that the tertiary structural context could expose the CDE stem more than expected at the experimental temperature of 37°C as compared to the other methods. Together, In-Line Probing and SHAPE provide valuable information on single-stranded regions and agree well with the NMR data. We thus postulate the secondary structure of the full-length *Ox40* 3′UTR as shown in Figure 3E where ADE and CDE adopt stable secondary structures. The Bulge as well as the central hinge region are flexible. Here, the low degree of secondary structure is well reflected by the alignment of *Ox40* 3′UTRs where we observe high conservation only within the same mammalian order (Supplementary Figure S6A and B).

### SAXS-derived 3D shapes of *Ox40* 3′UTR RNAs

Both the analysis of imino group NMR resonances and melting curves majorly focus on existing RNA base pairs, but they fail to report on the compactness of elements in different contexts. The solution method SAXS allows to derive geometric dimensions of a particle and can serve a basis for low-resolution models as excellently performed earlier with RNAs of different molecular weights and degrees of flexibility ((28,72), see e.g. SAXS applications).

We measured SAXS data from all RNA constructs of the *Ox40* 3′UTR and particularly focused on their derivable (Porod) volumes and maximum molecular extension D_max_ (Supplementary Figure S3C). Our data confirm the existence of folded RNA moieties as indicated by Kratky plots and respective distance distributions (Supplementary Figure S3C). Importantly, they reveal the absence of RNA element oligomers, which have shown to add another regulatory layer in a different study (73). Individual RNA element SAXS data allowed a juxtapositioning with RNA structural models created by *RNA composer* (74) or *RNAMasonry* (68). Indeed, we found a good fit between the experimental and back-calculated *D*_max_ values (Table 1). As expected, we observe larger *D*_max_ values than predicted for the RNAs containing the Bulge and flexible tail (full-length 3′UTR), which reflects the experimentally sampled conformational space, not representable in a single averaged model. However, the overall geometric dimensions are in excellent agreement with model-derived values and, most importantly, among themselves, respectively. For example, the summed-up experimental Porod volumes of ADE and Bulge (∼21 000 and 13 000 Å^3^, respectively) are well represented by the 34 000 Å^3^ seen for the Bulge-ADE tandem construct (Table 1). Further inclusion of the CDE (∼13 000 Å^3^) and Terminus (∼14 000 Å^3^) theoretically results in 61 000 Å^3^, which is excellently confirmed by the experimentally determined volume of 58 000 Å^3^ for the full-length 3′UTR. Likewise, the SAXS-derived molecular weights show the mere additive integration of monomeric RNAs into the full 3′UTR.

We finally calculated models of RNAs based on NMR derived base pairs and our SAXS curves using *RNAMasonry* (68). As shown in Supplementary Figure S3D, all models illustrate the NMR-supported secondary structure in a possible three-dimensionally sampled space. Of note, all structure models show low *X*^2^ values indicating their good agreement with the experimental SAXS data, and also for the full-length 3′UTR there is an excellent congruence of *D*_max_, *R*_g_ and derivable molecular weight of scattering data with the final structural model provided by *RNAMasonry*. Also, the models convincingly visualize the modularity of the *Ox40* 3′UTR with respect to its defined elements as the individual RNA elements excellently superimpose with a full-length 3′UTR model.

In sum, NMR, CD and SAXS data of available RNA constructs prove that the *Ox40* mRNA 3′UTR integrates individual folded RNA elements in an independent manner as shown for their two- and three-dimensional structures, as well as the individual thermal stabilities. In this regard, a full structure-guided picture of the 3′UTR shows a centrally placed and robust tandem of elongated ADE and CDE. The tandem is flanked by a flexible bulge with transient secondary structure and held together by a terminal kinked helix made up of long-range RNA interactions.

### Refolded RNAs resemble the native fold of the *Ox40* 3′UTR

We next sought to investigate the native *Ox40* 3′UTR in order to exclude structural artefacts from RNA refolding after purification. For small RNA elements like isolated stem–loops native-like folds can easily be obtained in most cases (75). However, a large complex folded RNA potentially involves different folding steps and kinetics including both fast and slowly folding elements (76). Thus, for the Bulge-ADE–CDE and the full-length 3′UTR constructs we purified RNAs via size-exclusion chromatography (SEC) in NMR buffer directly after transcription (Supplementary Figure S3E and F). Subsequent denaturing PAGE revealed excellent purity of the samples (Supplementary Figure S3E and F). NMR proton spectra of native samples were compared to their refolded analogues. For both the Bulge-ADE–CDE and full-length constructs, the respective spectral overlay provides virtually identical imino proton chemical shifts confirming the native-like state of refolded samples. We also investigated SEC profiles of native RNAs and found one prominent (>90%) and pure species for both of them. We found a small SEC peak preceding the main fraction of full-length 3′UTR, which yielded the same NMR spectrum, albeit with a lower signal-to-noise ratio. While excluding a dimer (see Table 1), we assume a low-populated second conformation in equilibrium with the main state, but averaged in NMR and SAXS. Overall, we here unambiguously confirm the native-like state of our refolded RNA constructs. This suggests they represent the *Ox40* 3′UTR fold *in vivo* with one major conformation.

### The contributions of individual RNA elements to Roquin-binding

The existence of extensive secondary structure within the *Ox40* 3′UTR poses the question for the role of the four structurally independent elements and their independence for recognition by MD-RBPs. Roquin had been found to bind the CDE and ADE elements through its unique core ROQ domain. However, previous studies failed to systematically investigate the role of the remainder (and major) sequence space within the *Ox40* 3′UTR. Additionally, the MD-nature of Roquin suggests possible roles for other domains apart from core ROQ.

Hence, we systematically probed all available RNA constructs for their capability to interact with the various domains of the structured Roquin amino-terminal part (N-term, residues 1–454, see Figure 1A). We did not observe binding of the RING domain to RNAs (not shown), supporting previous findings for RING as a protein-interacting domain (77,78). We thus focused on the established RBDs, i.e. core/extended ROQ and the ZnF, and purified a set of Roquin constructs integrating those RBDs as well as the full N-term (Supplementary Figure S4A, Table 2). We next employed electrophoretic mobility shift assays (EMSA) to quantify Roquin-RNA interactions. Notably, we did not observe mentionable binding of the Roquin ZnF to any of the structured RNAs or Tail RNA (Supplementary Figure S4), underlining its suggested preference for AU-rich ssRNA (56).

**Table 2.** SAXS-derived parameters for concentration series of different Roquin constructs. Theoretical molecular weights were obtained from (98). n.d. = not determined

Protein	Theoretical MW [kDa]	MW [kDa] / D_max_ [nm]			
		50 μM	100 μM	200 μM	300 μM
**ROQ**	17.77	16.77 / 5.15	15.47 / 5.65	18.05 / 6.07	19.92 / 7.07
**extROQ**	35.42	33.83 / 7.80	40.20 / 11.71	45.67 / 16.03	41.95 / 16.94
**N-term**	51.09	55.58 / 11.76	65.45 / 13.77	n.d.	94.23 / 28.30
**N-term A_mut_**	50.89	55.58 / 10.72	55.58 / 12.29	n.d.	67.08 /25.90
**N-term B_mut_**	50.96	58.15 / 11.26	59.50 / 14.72	n.d.	113.65 /21.90

**Figure 4.**
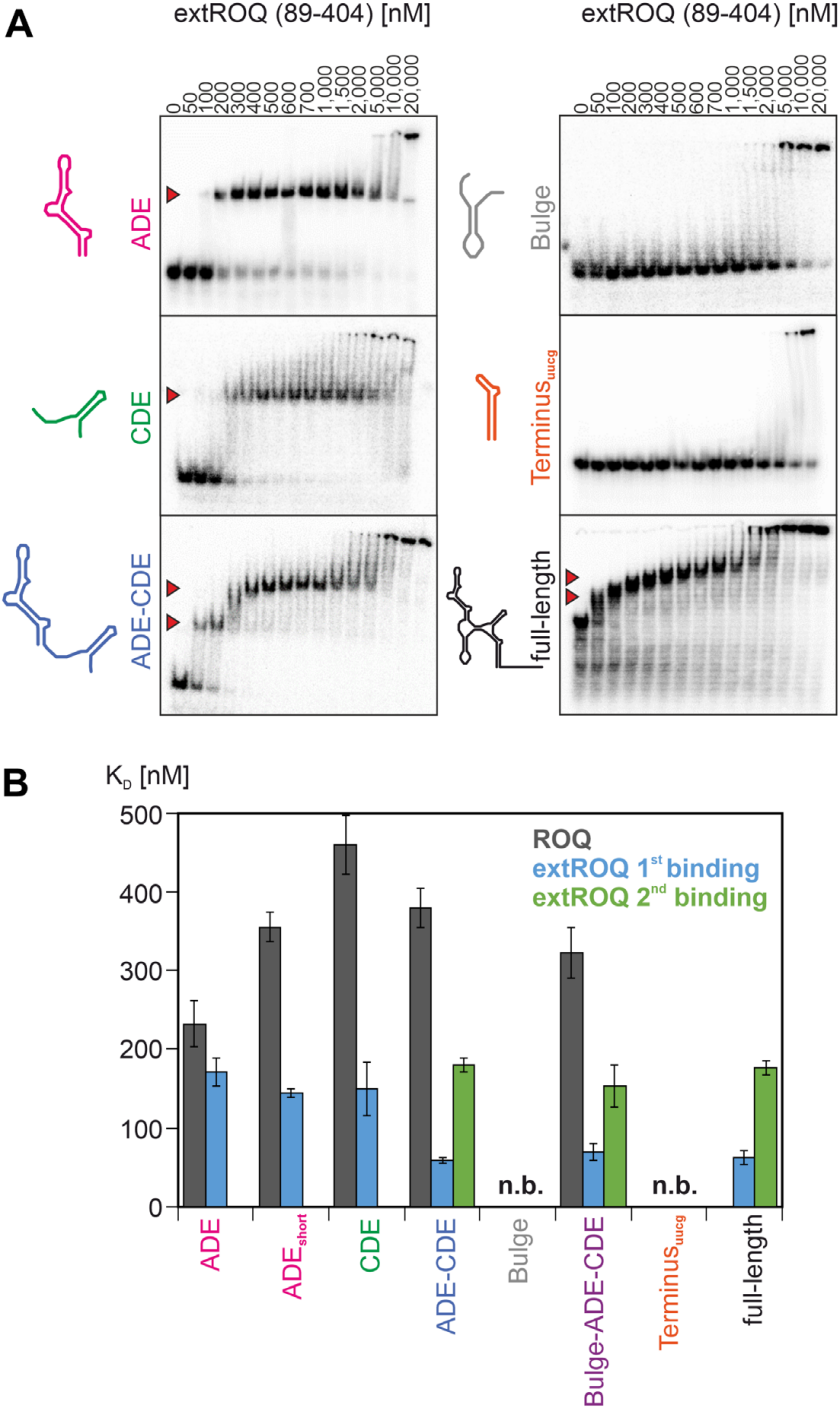
Binding of Roquin to *Ox40* 3′UTR RNAs. (**A**) EMSAs of extROQ with the depicted 3′UTR elements. Protein concentrations are given in nM on top. Red arrows indicate RNP complexes. (**B**) Quantification of EMSAs shown in (A) and Supplementary Figure S3. Grey represents binding of coreROQ, while blue and green depict first and second binding events of extROQ. n.b. = no binding.

Both the Roquin A- and B-sites had been described in the context of high-affinity binding to structured RNA. Based on that, we monitored binding of the extended ROQ domain (extROQ) to the *Ox40* elements by EMSA (Figure 4A). In line with earlier findings, we found nanomolar binding to the ADE and CDE, respectively, via a single transition (Figure 4B, Supplementary Figure S4, Table S4). We did not observe binding of the extROQ or core ROQ domains to the Bulge, Terminus or Tail RNAs for the applied concentrations of proteins. This is in agreement with our In-Line Probing data where we observed reduced cleavage in presence of Roquin only within the ADE and CDE loops (Figure 3D and E) confirming these as the sole Roquin binding sites. Altogether, we conclude that Roquin uses its extended ROQ domain to interact with the *Ox40* 3′UTR ADE–CDE—di-motif, while the other RNA elements and Roquin domains do not significantly contribute to complex formation of this regulatory mRNP.

### Binding of Roquin to the regulatory ADE–CDE core within the full-length *Ox40* 3′UTR

We next aimed to characterize the complex formation of Roquin and tandem ADE–CDE RNA in more detail. Earlier work had suggested the isolated core ROQ domain to bind the apical ADE_short_ with a preference over the CDE, supported by structural data (15). Compared to that, our EMSA data demonstrate a slightly increased affinity of core ROQ for ADE, indicating a role for the basal stem region in fine-tuning the complex formation. However, the ROQ domain still shows a clear preference for ADE over CDE (Figure 4B, Supplementary Figure S4C, Table S4), albeit both interactions are of nM-affinity. We next performed protein-detected NMR titrations of ROQ with either ADE or CDE RNAs (Supplementary Figure S4E) using 2D correlation spectra. We found characteristic amide resonances for ROQ that show differential chemical shifts upon RNA binding, which confirms specific recognition of the ADE hexa- or CDE tri-loops, respectively. In contrast, the extROQ domain exhibited no binding preference for CDE or ADE, despite a moderately increased affinity for both elements as compared to the core ROQ domain (Figure 4A, Supplementary Figure S4D, Table S4). We conclude that the Roquin B-site contributes to RNA binding in targeting the same molecule as the A-site, but through recognizing the double-stranded stem structure. This mechanistic feature had remained elusive so far (50) as the B-site interface was suggested to require a specific duplex length (50). Thus it is to our surprise that it enhances binding of the comparably short CDE stem loop (Supplementary Table S4). Interestingly, the ADE_short_ was bound by the extROQ domain with a comparable affinity as the ADE (Figure 4B, Supplementary Figure S4C) indicating the B-site interaction locates to the apical part. In agreement with that, In-line probing of full-length *Ox40* 3′UTR in complex with coreROQ revealed increased cleavage in stem regions of both ADE and CDE compared to a complex with extROQ (Figure 3D). This suggests stabilization of RNA stems through B-site interactions. Roquin binding, however, does not alter the secondary structure further and the 3′UTR retains its overall fold.

**Figure 5.**
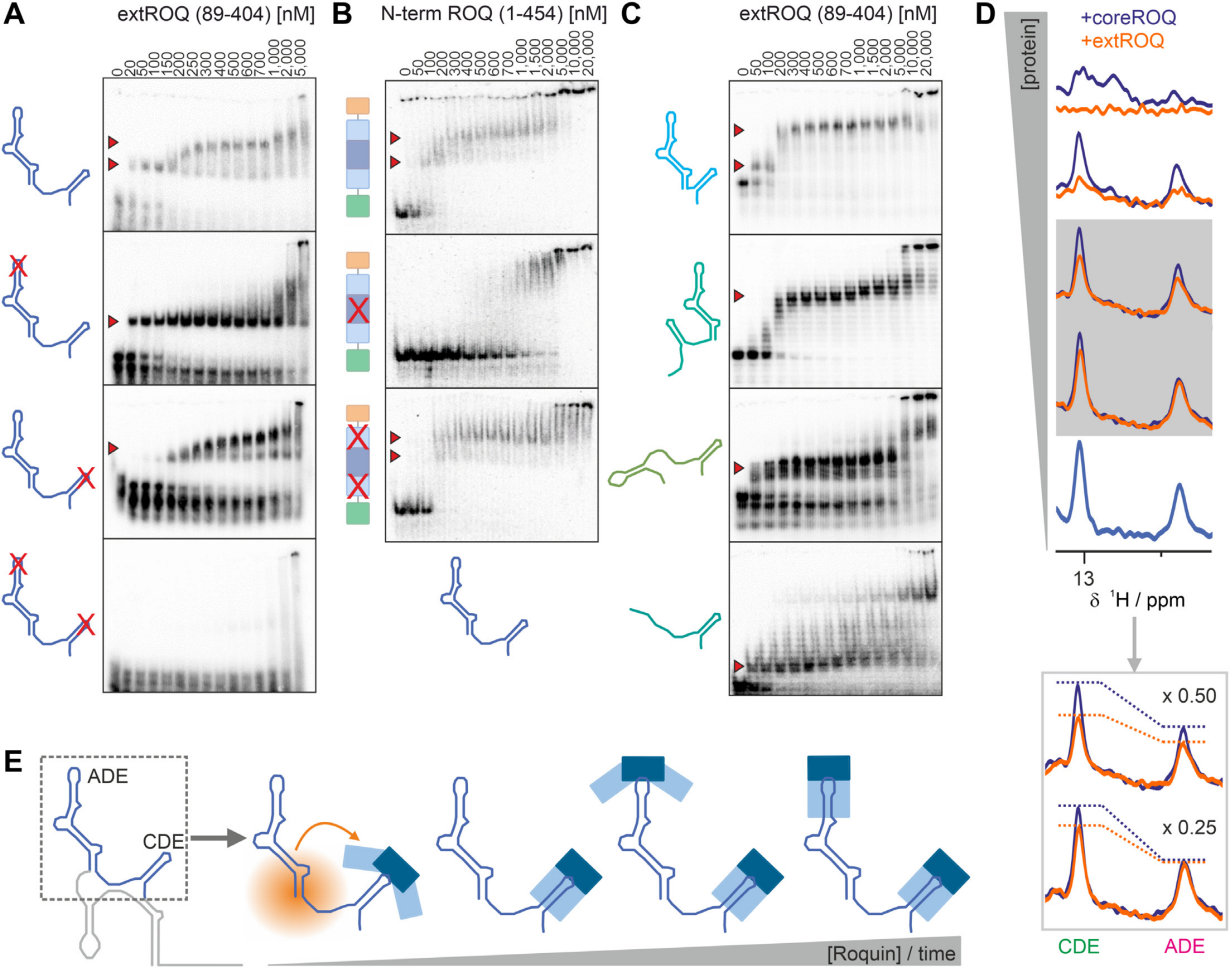
Sequential binding of two decay elements by Roquin. (**A**) EMSAs of extROQ with different WT and mutant versions of tandem ADE–CDE RNA. Protein concentrations are given in nM on top. Red arrows indicate binding events. (**B**) EMSAs of WT and mutant N-term Roquin with tandem WT ADE–CDE RNA. (**C**) EMSAs of extROQ with artificial CDE RNAs. (**D**) Excerpt of ^1^H imino spectral region of tandem ADE–CDE RNA alone and in presence of increasing concentrations of coreROQ or extROQ. The zoom-in highlights differential intensity changes at sub-stoichiometric protein levels (see Supplementary Figure S4). (**E**) Proposed model for the simultaneous and sequential binding of the ADE–CDE core within the *Ox40* 3′UTR by Roquin.

In the context of tandem ADE–CDE RNA, we consequently detected two distinct, quantifiable transitions for extROQ confirming a two-step binding of two Roquin molecules (Figures 4A and B, 5A, Supplementary Figure S4D). Likewise, we observed a 2:1 stoichiometry of extROQ interacting with the full-length 3′UTR, which is in line with EMSA data from isolated Bulge and Terminus that are incapable of binding Roquin. We also assume a 2:1 binding for core ROQ to tandem ADE–CDE and the full-length 3′UTR. While the two transitions were not resolvable by EMSA, NMR spectra of core ROQ complexed with tandem ADE–CDE yielded amide peaks with each one of the two chemical shifts characteristic for ADE or CDE (Supplementary Figure S4E). Alternatively, an intermediate chemical shift was present suggesting simultaneous binding of ADE and CDE. Interestingly, we observed more intense and slightly more peaks for the ADE-related signals confirming the moderate preference observed in EMSAs. Finally, and in line with data on extROQ, we also observe a two-step binding of the full Roquin N-term to the ADE–CDE motif (Figure 5B). As a result, the *Ox40* 3′UTR exclusively exploits its core ADE–CDE tandem for high-affine binding to Roquin, which in turn uses both its A- and B-sites for effective mRNP complex formation.

Given the 2:1 binding of Roquin to the *Ox40* 3′UTR, we asked whether dimerization of Roquin per se is a factor in complex formation with *Ox40* mRNA. Using SAXS experiments as a read-out for the proteins’ geometric parameters, we indeed found all Roquin constructs to dimerise in dependence of concentration (Table 2). However, for all of them significant contributions of dimers to the average molecular weight in equilibrium were only found at concentrations clearly above those used for EMSAs. Consequently, we do not consider Roquin dimers to be necessary for complex formation with *Ox40* 3′UTR RNAs.

### The roles of Roquin A- and B-sites for sequential complex formation with the 3′UTR

Binding of extROQ to decay elements within the ADE–CDE tandem occurs at approximately 59 and 180 nM (Figure 4A and B), and affinity of the first binding event is increased as compared to the isolated RNA elements. Interestingly, we did not observe this effect for the isolated core ROQ domain (Supplementary Figure S4C). We conclude that the increase in affinity is mediated through additional B-site interactions. We therefore set out to determine the order in which RNA elements are bound by Roquin. To this end, we created mutant versions of the ADE–CDE tandem RNA with either ADE, CDE or both stem loops mutated (Supplementary Figure S4B). The mutations had previously been shown to abolish protein binding based on nucleotide exchanges within the loops (15). We used imino proton-detected NMR to verify the structural integrity of the stem region (Supplementary Figure S5A). As expected, both individual ADE- and CDE-mutated tandems showed a single binding event in EMSAs, while the double mutant completely abolished binding to Roquin (Figure 5A). This suggests that RNP complex formation requires crucial interactions of the Roquin A-site with loop residues of decay elements. Further inspection of the characteristic complex band formation in EMSA gels suggests that the CDE is bound first in context of the tandem element (Figure 5A). This was confirmed by NMR titrations where we monitored line-widths of the RNA imino signals upon addition of either core ROQ or extROQ (Supplementary Figure S5B). We found uniform line-broadening of ADE and CDE resonances after adding sub-stoichiometric amounts of core ROQ (i.e. protein:RNA ratios ≤ 0.5), suggesting no preference in the binding order. In contrast, extROQ exhibited a stronger effect of line-broadening towards CDE resonances as compared to ADE signals. This observation suggests a small, but measurable preference for CDE-binding in the full-length Roquin context.

Our EMSA-based data suggested that the Roquin B-site alone does not mediate stable complex formation with RNA stem–loops itself. We asked whether the B-site can function in stabilizing the Roquin-3′UTR complex by increasing affinity, whereas the A-site accounts for specificity in the first instance. To address this experimentally, we mutated previously described key residues (54) in either the A-site or B-site of Roquin N-term (Figure 5B, Supplementary Figure S4A, Table S2). Of note, none of the mutations increased the tendency for the unbound N-term to dimerise as shown with SAXS data (Table 2). We then tested wildtype (WT) and mutant Roquin N-term for binding to the ADE–CDE tandem RNA. Identical to extROQ, we observed two binding events for the WT protein, while the A-site mutant did not interact with the RNA. This confirms that both RING domain and ZnF do not contribute to RNA-binding. It is also in line with the findings from RNA mutant experiments that underline A-site binding as a prerequisite for complex formation (Figure 5A). In contrast, the B-site mutant still showed a two-step binding, but with K_D_ values corresponding to isolated ADE and CDE elements. This corroborates a model where increased affinity for the CDE in presence of the ADE is mediated through the Roquin B-site.

### A 5′ RNA element enhances Roquin binding to the CDE

From the above, we speculated whether the overall RNA sequence or structure context itself contributes to Roquin affinity for the CDE. We systematically designed artificial RNAs to be tested in EMSAs (Supplementary Table S5). All RNAs were probed for structural integrity. NMR revealed the expected H-bond pattern (Supplementary Figure S5C-F), while SAXS confirmed the monomeric state of the RNAs. First, to examine the role of distance and conformational freedom between ADE and CDE, we removed the linker between the two decay elements (‘ADE-u-CDE’, Supplementary Table S5). We observed two-step binding comparable to WT ADE–CDE (Figure 5C) and conclude that 2:1 binding does not require full flexibility of the two elements. Interestingly, this finding reduces the likelihood of dimeric Roquin, which would require structural rearrangements of the RNA or not support available dimer models for the protein (50,55,79). Instead, the observation clearly supports two independent binding events.

To assess the role of the relative orientation of decay elements for the Roquin–CDE affinity, we next switched ADE and CDE (‘CDE–ADE’). Although the EMSAs still indicate two binding events, affinity decreases comparable to isolated elements. This clearly supports the model of two independent Roquin molecules binding to ADE–CDE. It, however, also suggests that an RNA element 5′ of the CDE is required for high affinity binding. With the Roquin B-site expected to have a major contribution to affinity, we next tested whether structured or duplexed RNAs are required in close 5′-proximity of the CDE. In a fusion construct (Bulge–CDE), we observed only a single binding event to the CDE which underlines Bulge is no Roquin target element (Figure 5C). Unexpectedly, affinity for the Bulge–CDE was significantly higher (∼50–100 nM) than for the CDE alone (∼150 nM), although the Bulge does not offer an extensive secondary structure for the extROQ. To rule out that the small double-stranded GC stretch of the Bulge is affecting affinity we fused the CDE to an unstructured RNA sequence (Supplementary Figure S5E) derived from *FARSA* mRNA 3′UTR (RefSeq entry NM_004461, (43)) (extCDE, Supplementary Table S5). Again, we detected a single binding event of extCDE to extROQ (approx. 50 nM) with an affinity similar to WT ADE–CDE. Altogether, our data suggest that any arbitrary single-stranded or structured RNA as to be found in a native context is capable of enhancing affinity for the CDE.

## DISCUSSION

The regulation of gene expression on the post-transcriptional level is majorly mediated through mRNA 3′UTRs, that are highly divergent in length, the content of structure and isoforms with relevance for transcript stability (8,13,80). Despite indication for a strong role in mRNA fate (81), we largely lack insights into 3′UTR structure and plasticity as structural characterization of large RNAs remains a major challenge. Absence or masking of 3′UTR *cis* elements related to RNA structure can shift the engagement with regulatory RBPs or microRNAs (47,82–85). Evidence of a highly structured 3′UTR landscape (12) has made structural characterization of expanded RNA contexts a prerequisite for mechanistic insights into regulatory mRNPs, but has not been approached for 3′UTRs with high-resolution to date.

### The *Ox40* 3′UTR is highly structured, but comprises majorly independent RNA elements

We here show that a divide-and-conquer approach enables structural analysis of a full-length mRNA 3′UTR of 157 nt in size. The approach was guided by secondary structure prediction and the production of single and combined RNA fragments analysed by nt-resolved NMR and supported by SAXS. Previous studies (30) and the present approach suggest that multiple small RNA fragments provide a possible shortcut to initial secondary structure models and the rational design of more complex RNAs. Our NMR analysis of individually predicted RNA elements confirmed the proposed overall *Ox40* 3′UTR structure (Figure 1) as organized in four domains with extended long-range interactions between the 3′UTR ends. As such, the 3′UTR represents a self-contained RNA hub, whose structure requires the complete sequence. The role of full 3′UTR context for functional efficacy has been a matter of debate for long (8). Our experimentally derived secondary structure provides a paradigm of how to overcome ambiguous findings from non-identical sequence contexts, as shown before (86).

Among the RNA domains, we found the previously described ADE and CDE stably embedded in a larger structured context. Of particular interest, we identified the original ADE stem–loop to be the apical part of an elongated, kinked stem. A dynamic region within the ADE stem would principally allow formation of low-populated conformations. In contrast, the CDE adopts one well-defined structure, and our NMR data prove the CDE to be present independent of various larger contexts (Figure 3). Of note, an alignment of *Ox40* 3′UTRs across vertebrates reveals a highly conserved sequence distance between the ADE and CDE elements despite obviously less conserved flanking regions in the respective UTRs (Supplementary Figure S6A and B). This hints at co-evolutionary function of the two elements and is supported by their central role for both the overall UTR structure and engagement with Roquin in this study. The NMR data also reveal additional, non-linear RNA elements. Their broadened NMR resonance linewidths indicate higher flexibility and coincide with lower thermal stability from CD melting curves. Our data prove e.g. that the Bulge, even though predicted to be stably base-paired, is a low-structure RNA region. Secondary structure predictions of other mammalian *Ox40* 3′UTRs confirm the region 5′ of the ADE to be indeed flexibly structured (Supplementary Figure S6C).

The combination of multiple 3′UTR elements revealed, by individual base pair-resolved NMR, that all four domains are independent and no interactions occur between them. Two points lead to this conclusion: First, imino group NMR resonances of combinatory RNA elements exhibit no or minimal chemical shift differences compared to isolated ones (Figure 3). Second, line-widths of the fragments differ significantly among each other, also in the context of the full-length 3′UTR, indicating individual movement enabled by flexible linkers rather than a common tumbling. This is further supported by the inclusion of our SAXS data, which underline a modular arrangement of RNA elements within the full UTR. In this, we were able to use excellent 3D models of RNAs as proven by their fit with individual SAXS scattering profiles (Supplementary Figure S3). Of note, the combined utilization of NMR (including full experimental structures) and SAXS data has very recently allowed determining a structural ensemble of the Neurospora Varkud Satellite ribozyme of >100 nt with an excellent convergence/resolution (87), which underlines the strong potential of combining the two solution methods. Finally, our In-line probing and SHAPE experiments confirm the preservation of secondary structure of individual RNA elements within the entire 3′UTR. In a similar study, Brunel *et al.* discovered that the 3′UTR of *bicoid* mRNA is composed of independently folded domains connected by a flexible central hinge region (88). Both cases raise the question whether structurally uncoupled *cis* elements are the functional active form in 3′UTRs or whether they require interactions with one another. For *Ox40* the high conservation of sequence and structure suggests that independence within the UTR is an evolutionary conserved feature (Supplementary Figure S6). In several cases long-range RNA-RNA interactions have been described as crucial, e.g. for cyclization of viral RNA genomes (89). However, only few studies analysed intramolecular interactions between folded *cis* RNA elements of a 3′UTR. In fact, those studies are often biased toward independently folded and acting *cis* elements by prediction, as e.g. for the well-studied *Hmga2* (9). Additional elements are not considered and experimentally examined. For others, e.g. *P5abc*, both independent and cooperative folding of elements was observed (90).

We claim, that inherent RNA dynamics and poor folding predictability demand for an empirically driven structure-probing of expanded 3′UTR contexts at high resolution to examine the full *cis* repertoire and its capability in RNP formation. Here, despite their independence in the apo form, RNA elements may still concert in the formation of Roquin RNPs, e.g. by steric hindrance. The latter point is of relevance e.g. for the four-domain N-terminus exploited in this study. It would also be of relevance considering potential RBP oligomers binding to target RNA. Despite little evidence for functionally relevant dimers of Roquin from SAXS, our data leave room for speculation about Roquin dimerization upon 3′UTR-binding. Such scenario could e.g. affect downstream processing of the target mRNA by enhanced, avidity-driven recruitment of the decay machinery.

### A spatiotemporally resolved engagement of the RBP Roquin with the *Ox40* 3′UTR

The full 3′UTR secondary structure enabled us to examine all RNA elements for binding to individual Roquin domains. We show that core ROQ, the extROQ and the full N-terminus bind ADE and CDE (Figure 4) with high affinity in all RNA contexts. Neither the Bulge nor the Terminus showed a detectable effect on protein binding suggesting these elements remain independent also in complex with Roquin. However, they might play a role in the regulation of *Ox40* mRNA by other RBPs. Likely, the respective combination of RBDs and *cis* elements is a major parameter of individual mRNA regulation. In line with that, we found no contribution of the Roquin ZnF to RNP formation with the *Ox40* 3′UTR, although the domain was shown to be of relevance in the binding to *ICOS*, *A20* and *NFkbiz* ssRNA regions (48,56,91).

We find that the presence of an arbitrary RNA in 5′ of the CDE enhances binding to Roquin (Figure 5). Although this enhancement is well-mediated by the ADE, it effectively does not require RNA structure. Our data suggest that the Roquin B-site is attracted to the 3′UTR by negative charges and thereby facilitates specific complex formation through A-site binding. This is the first example of a neighbouring element to modulate CDE-recognition by Roquin but had been experimentally indicated for a different stem–loop in *A20* mRNA before (48). It highlights the importance of studying regulatory *cis*-elements in their native structural context. Further, the interplay of multiple *cis*-elements and their subtle differences in affinity or stability, e.g. between ADE and CDE, will only be of relevance in a holistic context. Of note here, the B-site is supportive for the specific complex formation of core ROQ with a stem–loop. We propose a two-step binding mechanism where the core ROQ domain initiates complex formation by recognizing a loop structure through its A-site before the B-site locks the complex through interactions with the RNA backbone. Such mechanism allows fine-tuning of *cis*-element recognition and offers an explanation for the inconsistent length of stems in ADEs and CDEs throughout different 3′UTRs (43). Furthermore, larger stems will lead to exposure of loops, facilitate Roquin binding and might support binding of lower-affinity elements like the previously described LBE RNAs (56).

The role of more than one decay element within 3′UTRs remains elusive and an important question to address in the future, not exclusively for Roquin-regulated mRNAs. Multiple decay elements might lead to a simple increase in efficacy of mRNA decay governed by avidity in RBP-binding. Similarly, multiple elements could steer mRNA turnover through simultaneous cofactor binding. Such *trans* cofactors will potentially modulate Roquin sequestration, as e.g. recently shown for NUFIP2 (23), and they have also been found for other RBPs (92). Depending on their spatiotemporal abundance proteins will compete for target binding with opposing or redundant effects (93,94) by using identical or selective *cis* elements in one mRNA. The subtle differences in affinity we detect e.g. between *Ox40* ADE and CDE could favour binding of one protein or the other as we have recently shown for the competition of Roquin and AUF1 for AREs (47). To completely understand control of mRNA decay, the interactions of the *Ox40* 3′UTR with co-existing *trans* factors, e.g. Regnase and Arid5a, will need to be dissected in an analogous manner as for Roquin. Regnase as a ribonuclease is required together with Roquin to cooperatively degrade mRNAs (95) while Arid5a positively effects mRNA stability in T cells (96). All three proteins target structured RNA within the *Ox40* 3′UTR, which underlines its paradigmatic nature as a mRNA regulatory hub and was the rational basis for its choice in this study. Future studies will have to unravel their compulsory effects on 3′UTR structure and occupation in a complete regulatory RNP to showcase integrated mRNA decay control. Our detailed, high-resolution description of the *Ox40* 3′UTR secondary structure and its role for engagement with the MD-RBP Roquin serves a first step towards a comprehensive description of this hub by structural and functional means.

## DATA AVAILABILITY

NMR chemical shifts of *Ox40* mRNA 3′UTR imino group ^1^H and ^15^N nuclei have been deposited at the BioMagResBank (BMRB) under ID 50846. SAXS scattering data of all RNA elements have been deposited at the SASDB under IDs: SASDNW5, SASDNX5, SASDNY5, SASDNZ5, SASDN26, SASDN36, SASDN46, SASDN56, SASDN66, SASDN76, SASDN86, SASDN96, SASDNA6, SASDNB6, SASDNC6. Illumina sequencing data from SHAPE-seq experiments are accessible under SRA/BioProject with ID: PRJNA816904, or accessible via https://www.ncbi.nlm.nih.gov/bioproject/816904.

## Supplementary Material

gkac212_Supplemental_File
