# Supplementary material for: NMR-derived secondary structure of the full-length Ox40 mRNA 3′UTR and its multivalent binding to the immunoregulatory RBP Roquin
Source: Nucleic Acids Res. 2022 Mar 31;50(7):4083–99. doi: 10.1093/nar/gkac212 (PMC9023295; doi:10.1093/nar/gkac212)
Supplement: gkac212_Supplemental_File [file gkac212_supplemental_file.pdf]

Supplementary information

NMR-derived secondary structure of the full-length *Ox40* mRNA 3'UTR and its multivalent binding to the immunoregulatory RBP Roquin

Jan-Niklas Tants[1], Lea Marie Becker[1], François McNicoll[2], Michaela Müller-McNicoll[2], Andreas Schlundt[1,*]

[1]Goethe University Frankfurt, Institute for Molecular Biosciences and Biomagnetic Resonance Centre (BMRZ), Max-von-Laue-Str. 9, 60438 Frankfurt, Germany

[2]Goethe University Frankfurt, Institute for Molecular Biosciences, Max-von-Laue-Str. 13, 60438 Frankfurt, Germany

* To whom correspondence should be addressed. Tel: +49 69 798 29699; Fax: +49 69 798 29225; Email: schlundt@bio.uni-frankfurt.de

Supplementary Table S1: Sequence boundaries of wildtype *Ox40* 3'UTR RNA constructs of this study.

RNA	Residues	Nucleotides
**ADE**	52-100	49
**ADE_{short}**	g|67-87	22
**CDE**	98-126	29
**CDE_{short}**	109-123	15
**ADE-CDE**	52-126	75
**Bulge**	17-51	35
**Bulge-ADE**	17-100	84
**Bulge-ADE-CDE**	17-126	110
**$Terminus_{UUCG}$**	1-13|uucg|126-140	32
**Full-Length**	1-157	157

Supplementary Table S2: Protein constructs of this study. Residue numbers refer to reference (1).

Protein	Residues	Theoretical MW [kDa]	Mutations
**ROQ**	171-326	17.77	-
**extROQ**	89-404	35.42	-
**N-term**	1-454	51.09	-
**N-term A_{mut}**	1-454	50.89	K220A, K239A, R260A
**N-term B_{mut}**	1-454	50.96	R135E, K136E, D322A, K323A
**ZnF**	411-454	5.24	-

Supplementary Table S3: Primers used to generate IVT templates for native RNA purification.

Primer	Sequence (5'→3')
**Forward**	CAG CTA TGA CAT GAT TAC GAA TTC
**Reverse full-length**	TAG CAT GTT TAT TAG GAG CAC C
**Reverse CDE**	GTA GTA TGC ATA GCA TAC ATA GGA AAG

Supplementary Table S4: Affinities of Roquin constructs for different *Ox40* 3'UTR RNA fragments. K_D values are given in nM; asterisks indicate triplicates. n.b. = no binding

Protein / RNA	ROQ	extROQ	N-term	N-term A_{mut}	N-term B_{mut}
**ADE**	232 ± 29*	171 ± 17*	-	-	-
**ADE_{short}**	355 ± 18	144 ± 6	-	-	-
**CDE**	460 ± 37*	149 ± 34*	-	-	-
**ADE-CDE**	379 ± 25	59 ± 4<br>180 ± 9	78 ± 4<br>297 ± 53	593 ± 23	149 ± 10
**Bulge**	3077 ± 634	1139 ± 81	-	-	-
**Bulge-ADE-CDE**	323 ± 32	69 ± 11<br>153 ± 26	-	-	-
**$Terminus_{UUCG}$**	-	n.b.	n.b.	-	-
**Full-Length**	-	63 ± 9	-	-	-

Supplementary Table S5: Sequences of artificial RNA constructs based on the *Ox40* 3'UTR. Underlined residues derive from the *FARSA* mRNA 3'UTR. Bold residues highlight primer binding site for reverse transcription.

RNA	Sequence (5'→3')
**ADE-u-CDE**	GCCUGCCAGUACCCUCCACACCGUUCUAGGUGCUGGGCUGGCUCUGGGCUGUA<br>UGCUAUGCAUAC<br>UAC
**CDE-ADE**	GGUUUCCUAUGUAUGCUAUGCAUACUACUUUCCGCCUGCCAGUACCCUCCACAC<br>CGUUCUAGGUG<br>CUGGGCUGGCUCUGGGC
**Bulge-CDE**	GGAUUUUAUGGGGCACGGACAACCCAUAUCCUGAUUUUCCUAUGUAUGCUAUGC<br>AUACUAC
**extCDE**	GUAUUUAUGAUUUCCUAUGUAUGCUAUGCAUACUAC
**full-length SHAPE**	GCAUUACUACAGGAGUGGAUUUUAUGGGGCACGGACAACCCAUAUCCUGAUGCC<br>UGCCAGUACCCUCCACACCGUUCUAGGUGCUGGGCUGGCUCUGGGCUUUCCUA<br>UGUAUGCUAUGCAUACUACCUGCCUGGUGGUGCUCCUAAUAAACAUGCUA**GAUC**<br>**GGAAGAGCACACGUCUG**

Supplementary Table S6: Primers used for library generation for Shape-Seq. Underlined residues indicate experimental barcodes, bold residues random barcodes.

Primer	Sequence (5'→3')
UDI0001_Fwd	CAAGCAGAAGACGGCATACGAGATCCGCGGTTGTGACTGGAGTTCAGACGTGTGCTCTTCCGATC
UDI0002_Fwd	CAAGCAGAAGACGGCATACGAGATTTATAACCGTGACTGGAGTTCAGACGTGTGCTCTTCCGATC
UDI0003_Fwd	CAAGCAGAAGACGGCATACGAGATGGACTTGGGTGACTGGAGTTCAGACGTGTGCTCTTCCGATC
UDI0004_Fwd	CAAGCAGAAGACGGCATACGAGATAAGTCCAAGTGACTGGAGTTCAGACGTGTGCTCTTCCGATC
UDI0005_Fwd	CAAGCAGAAGACGGCATACGAGATATCCACTGGTGACTGGAGTTCAGACGTGTGCTCTTCCGATC
UDI0006_Fwd	CAAGCAGAAGACGGCATACGAGATGCTTGTCAGTGACTGGAGTTCAGACGTGTGCTCTTCCGATC
UDI0001_Rev	AATGATACGGCGACCACCGAGATCTACACAGCGCTAGACACTCTTTCCCTACACGACGCTCTTCCGATCT**NNNNN**GCATTACTACAGGAGTGGATTTTATGG
UDI0002_Rev	AATGATACGGCGACCACCGAGATCTACACGATATCGAACACTCTTTCCCTACACGACGCTCTTCCGATCT**NNNNN**GCATTACTACAGGAGTGGATTTTATGG
UDI0003_Rev	AATGATACGGCGACCACCGAGATCTACACCGCAGACGACACTCTTTCCCTACACGACGCTCTTCCGATCT**NNNNN**GCATTACTACAGGAGTGGATTTTATGG
UDI0004_Rev	AATGATACGGCGACCACCGAGATCTACACTATGAGTAACACTCTTTCCCTACACGACGCTCTTCCGATCT**NNNNN**GCATTACTACAGGAGTGGATTTTATGG
UDI0005_Rev	AATGATACGGCGACCACCGAGATCTACACAGGTGCGTACACTCTTTCCCTACACGACGCTCTTCCGATCT**NNNNN**GCATTACTACAGGAGTGGATTTTATGG
UDI0006_Rev	AATGATACGGCGACCACCGAGATCTACACGAACATACACACTCTTTCCCTACACGACGCTCTTCCGATCT**NNNNN**GCATTACTACAGGAGTGGATTTTATGG

Supplementary Table S7: Primer combinations used for library generation for Shape-Seq.

Sample	Forward primer	Reverse primer
**DMSO rep. 1**	UDI0002_Fwd	UDI0002_Rev
**DMSO rep. 2**	UDI0005_Fwd	UDI0005_Rev
**NMIA rep. 1**	UDI0001_Fwd	UDI0001_Rev
**NMIA rep. 2**	UDI0004_Fwd	UDI0004_Rev
**1M7 rep. 1**	UDI0003_Fwd	UDI0003_Rev
**1M7 rep. 2**	UDI0006_Fwd	UDI0006_Rev

Supplementary Figure S1:

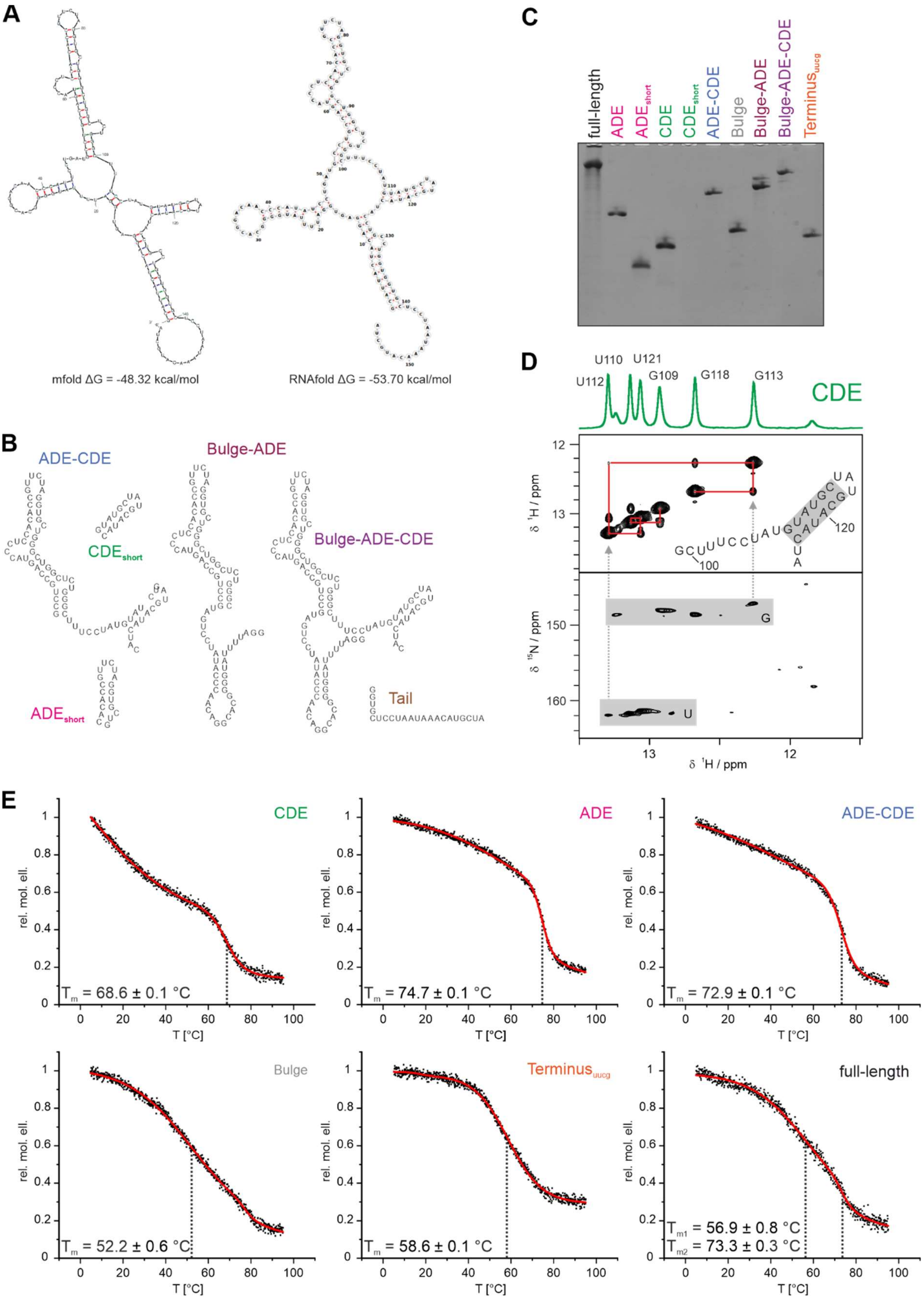

Supplementary Figure S1. Biophysical characterization of *Ox40* 3'UTR RNA elements. **(A)** Secondary structure predictions of full-length *Ox40* 3'UTR using *mfold* (2) and *RNAfold* (3). **(B)** Secondary

structures of fragments predicted from full-length 3'UTR used in this study. **(C)** Denaturing PAGE showing all RNA fragments. **(D)** ^{1}H,^{1}H-NOESY and ^{1}H,^{15}N-SOFAST-HMQC of CDE RNA. Red lines indicate sequential assignments. Gs and Us are indiacted within their typical chemical shift ranges in the 2D ^{1}H,^{15}N correlation spectrum. **(E)** CD melting curves of RNA constructs with fits and calculated melting temperatures.

Supplementary Figure S2:

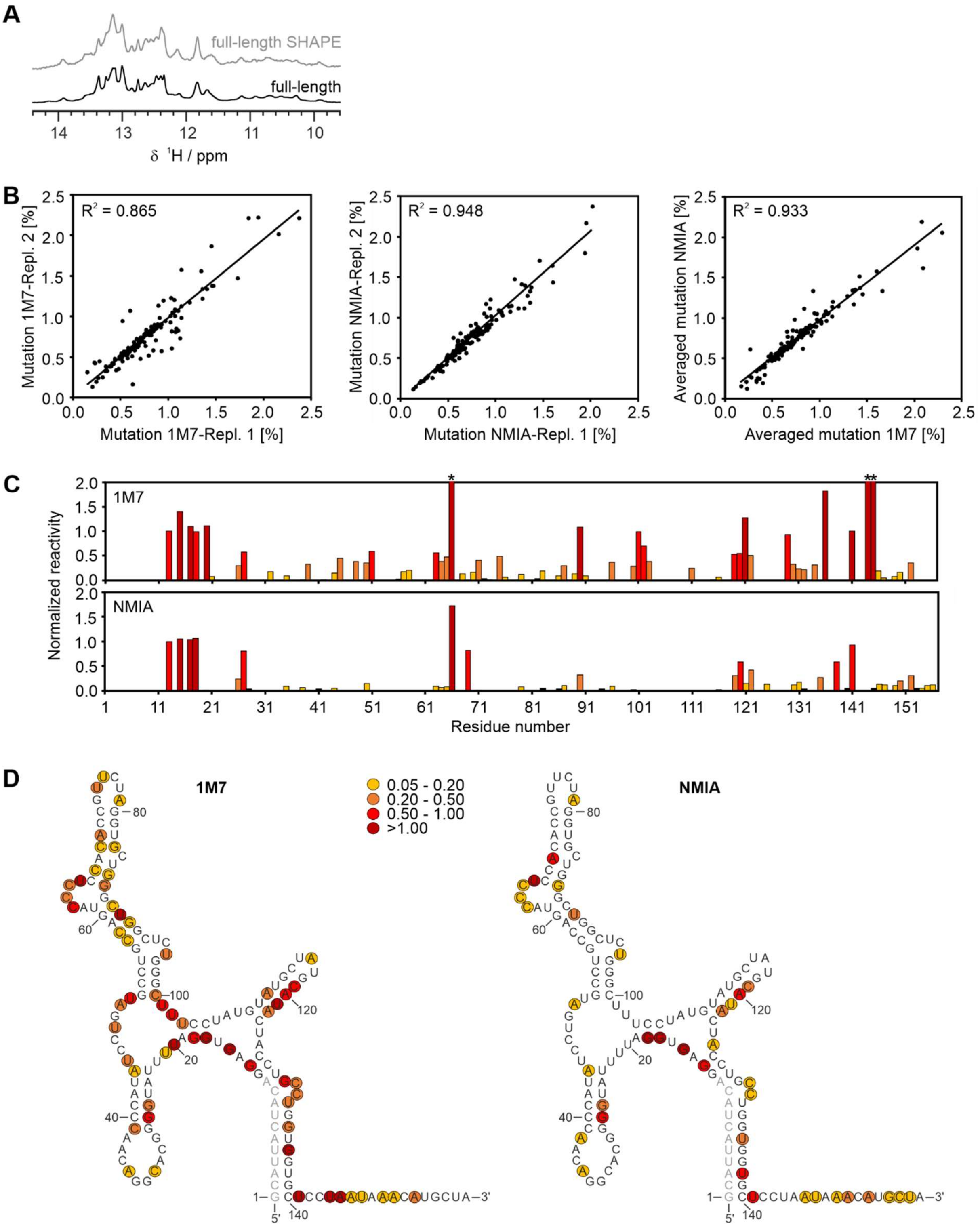

Supplementary Figure S2. SHAPE analysis of full-length *Ox40* 3'UTR. **(A)** Imino ^{1}H proton spectra of full-length *Ox40* 3'UTR and the 'full-length SHAPE' RNA variant containing the RT primer binding site shown in Supplementary Table S5. **(B)** Correlation plots of SHAPE replicates using 1M7 and NMIA showing the mutation rate in percent. On the right the correlation between both chemicals is plotted. **(C)** Normalized SHAPE reactivity of averaged replicates for 1M7 and NMIA per residue of *Ox40* 3'UTR. Bars are colored according to reactivity shown in (D). Asterisks indicate residues with reactivity > 2. **(D)** SHAPE reactivities for both chemicals plotted onto secondary structure. Reactivity is color coded as indicated.

Supplementary Figure S3:

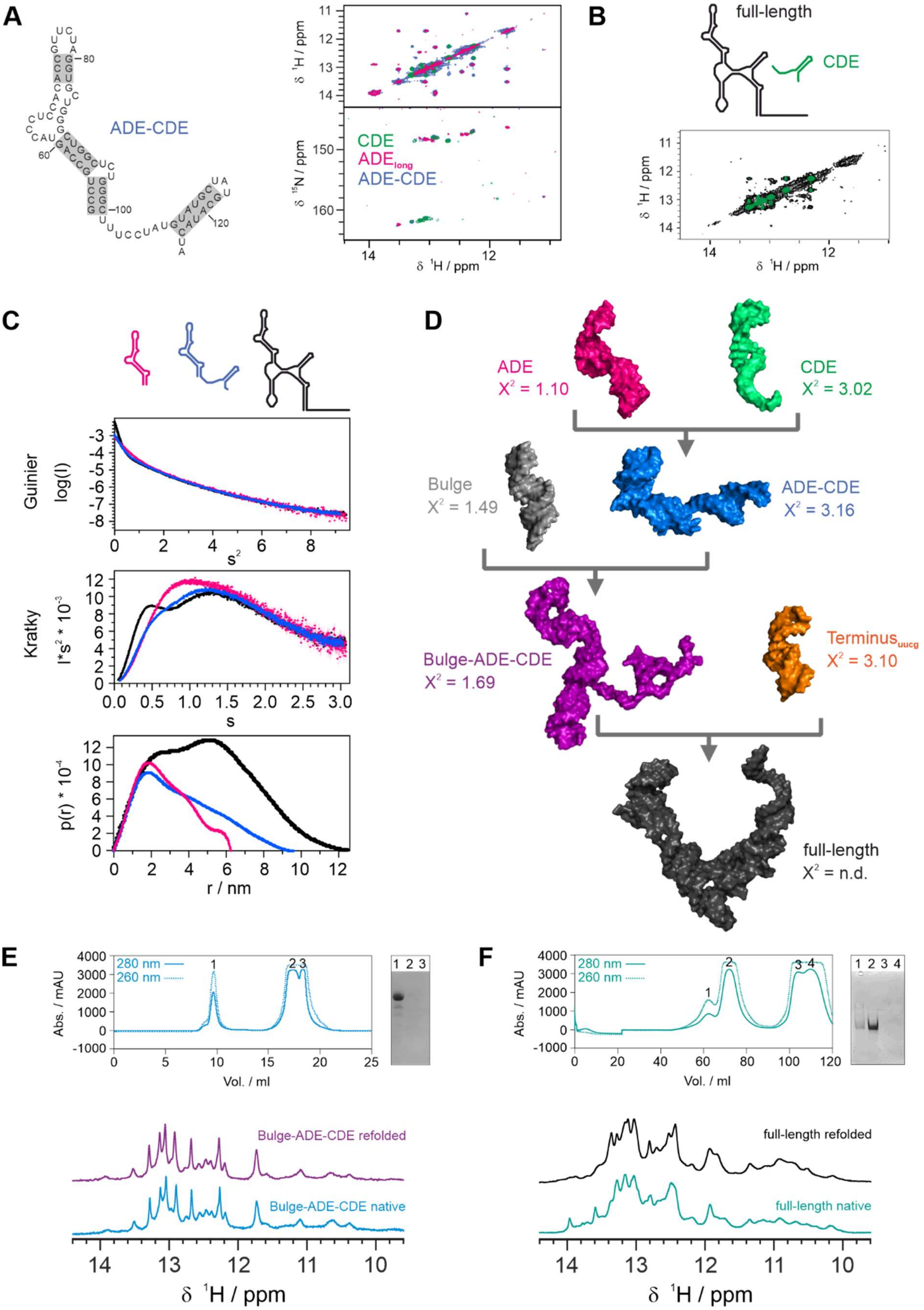

Supplementary Figure S3. SAXS analysis of *Ox40* 3'UTR RNA fragments. **(A)** Secondary structure prediction of the ADE-CDE fragment. Grey boxes indicate confirmed base pairs based on $^{1}H,^{1}H$-NOESY

and ^{1}H,^{15}N-SOFAST-HMQC spectra on the right, as well as assignments of ADE- and CDE-only RNAs (see Figure 1). **(B)** Overlay of ^{1}H,^{1}H-NOESY spectra of CDE only (green) and *Ox40* full-length 3'UTR (black). Secondary structure predictions are shown as cartoons above. **(C)** SAXS data of ADE, tandem ADE-CDE and full-length 3'UTR. From top to bottom: Guinier plot, Kratky plot and P(r) curves. The Guinier plot shows initial linearity and high signal-to-noise levels while the Kratky plot confirms the folded state of all RNAs. The D_{max} can be determined from the P(r) curve. **(D)** Surface depiction of SAXS-based models of the four elements, the ADE-CDE and Bulge-ADE-CDE RNA and the full-length 3'UTR. Chi^2 values (X^2) are derived from fitting the models to experimental SAXS curves using CRYSOL (4). Note that no Chi^2 was determined for full-length *Ox40* 3'UTR, but the validity of the model relative to the scattering data is shown via D_{max}, R_g and the MW in main text **Table 1**. *RNAMasonry* (5) was used to generate models. The flow scheme visualizes the modularity of the *Ox40* 3'UTR. **(E)** Size-exclusion UV chromatogram of Bulge-ADE-CDE and denaturing PAGE of collected fractions as indicated. Dashed lines show absorption at 260 nm, solid lines at 280 nm. The corresponding imino ^{1}H spectral region of fraction 1 is shown at the bottom (blue) compared to a refolded sample (purple). **(F)** Same experiment as in (E) for the full-length 3'UTR. The spectra of fraction 1 and 2 are identical; only the spectrum of fraction 2 is shown for a better S/N.

Supplementary Figure S4:

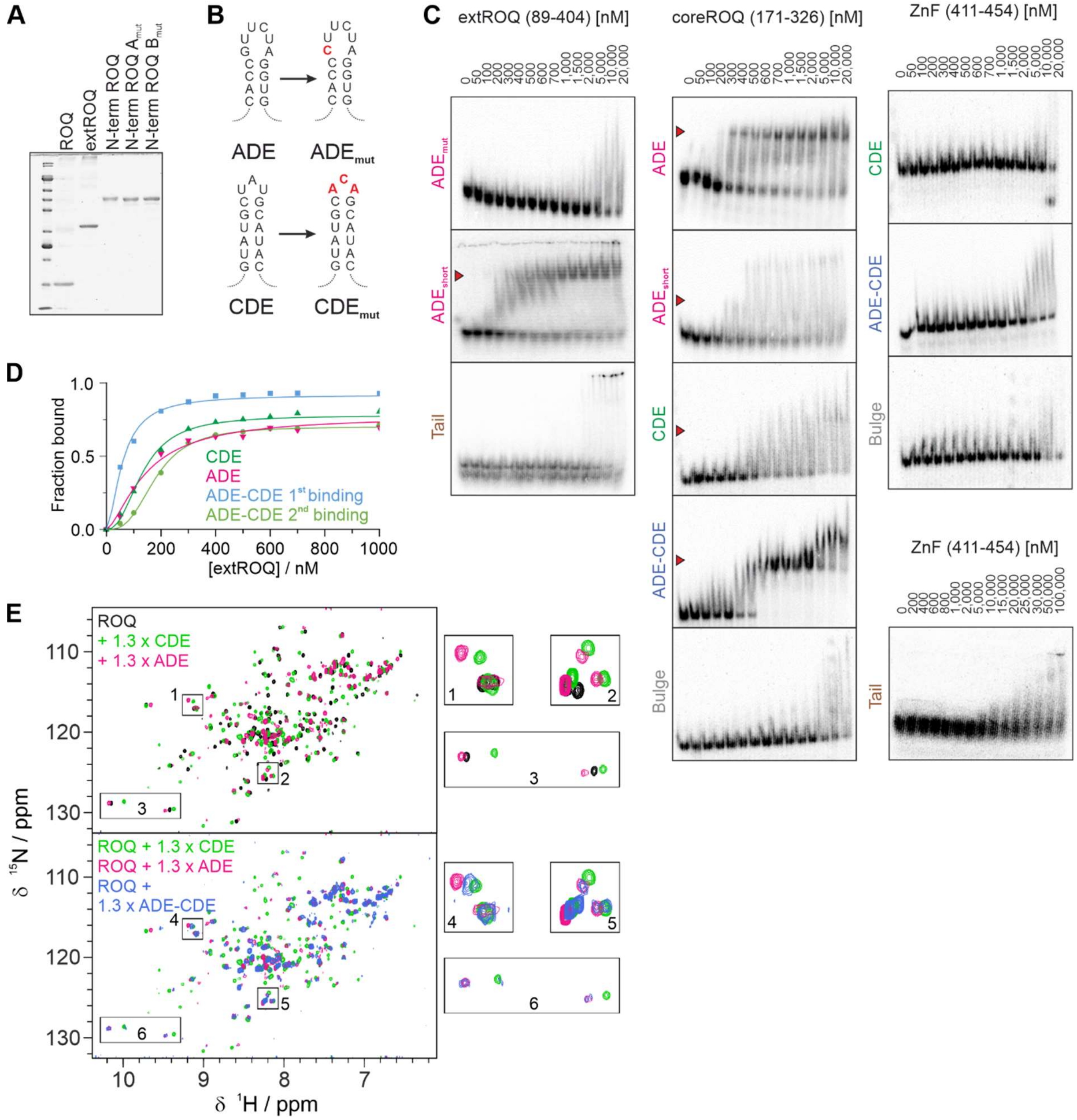

Supplementary Figure S4. Binding of Roquin domains to *Ox40* 3'UTR fragments. **(A)** SDS-PAGE of protein constructs as used in this study. (**B**) Schematic depiction of ADE and CDE wildtype and mutant sequences in its fold used for binding experiments. **(C)** Electrophoretic mobility shift assays of core ROQ, ZnF and extROQ with fragments of the *Ox40* 3'UTR. Red arrows indicate binding events. **(D)** EMSA-based fit of extROQ binding to ADE, CDE and tandem ADE-CDE. **(E)** $^{1}H,^{15}N$-HSQC spectra of free coreROQ (black), in complex with CDE (green) or ADE (magenta) (top panel) and in complex with CDE, ADE and tandem ADE-CDE in green, magenta and blue, respectively (bottom panel). Numbered boxes are shown as zoom-ins on the right.

Supplementary Figure S5:

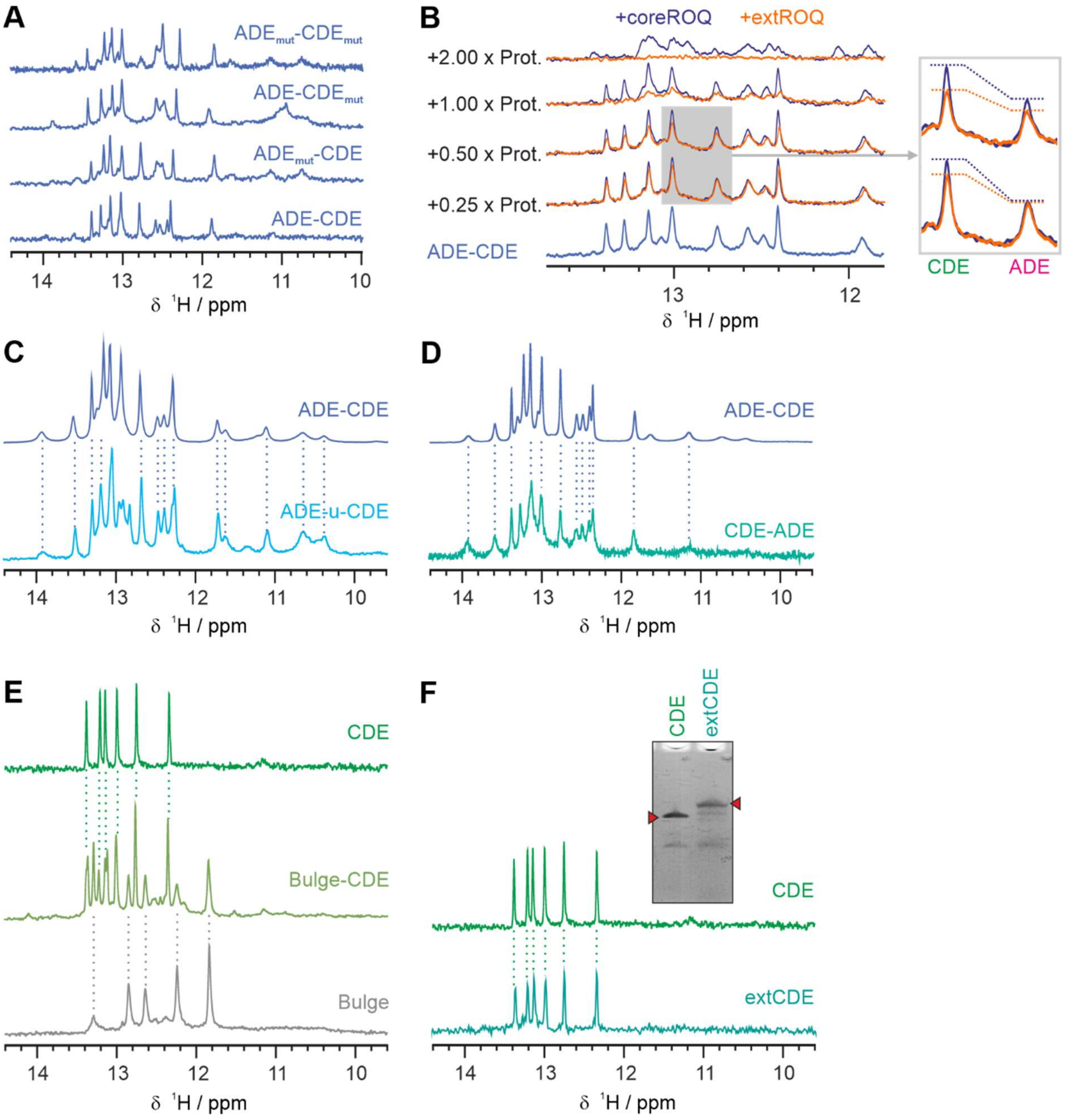

Supplementary Figure S5. Probing secondary structures of RNA variants and binding preferences by NMR. **(A)** Imino proton spectra of RNA mutants in the ADE-CDE tandem construct context. **(B)** Imino proton spectra of free tandem ADE-CDE alone and with increasing amounts of coreROQ (blue) or extROQ (orange). The grey box indicates the zoom-in shown on the right to highlight a comparison of CDE and ADE related resonances. **(C-F)** Imino proton spectra of artificial RNAs compared to the respective reference wildtype spectrum. **(F)** additionally shows the PAGE of extCDE compared to wildtype CDE.

Supplementary Figure S6:

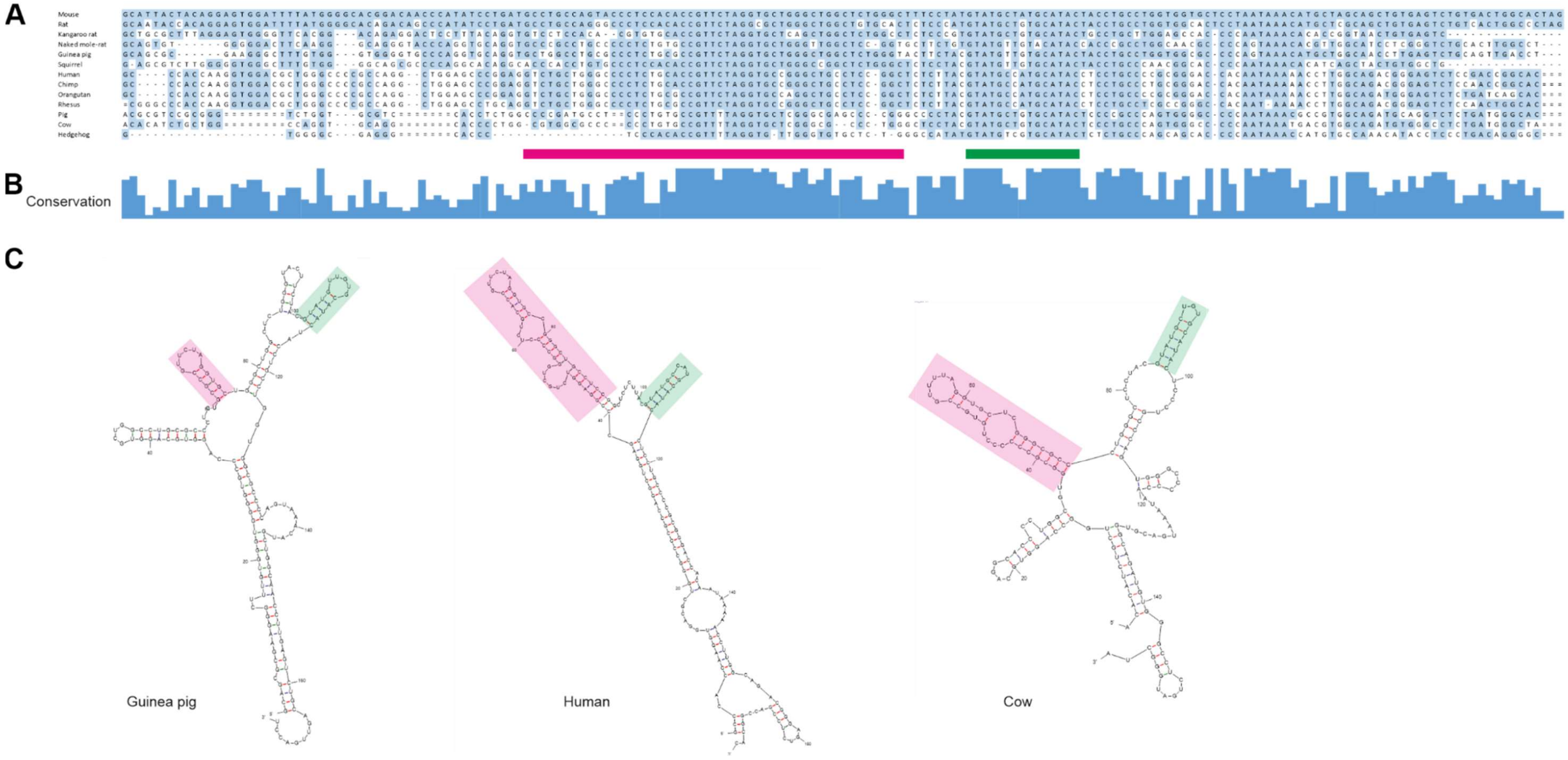

Supplementary Figure S6. Structural conservation of *Ox40* 3'UTR elements. **(A)** *Ox40* 3'UTR *Multiz* Alignment (6-8) of selected mammals. The magenta and green bars below indicate structured ADE and CDE elements determined for murine *Ox40* 3'UTR in this study. **(B)** Conservation of individual positions based on alignment shown in (A), **(C)** Secondary structure predictions of guinea pig, human and bovine *Ox40* 3'UTR based on sequences shown in(A). For predictions *mfold (*2) was used. Colored boxes indicate ADE and CDE elements.

SUPPLEMENTARY REFERENCES

1. Essig, K., Kronbeck, N., Guimaraes, J.C., Lohs, C., Schlundt, A., Hoffmann, A., Behrens, G., Brenner, S., Kowalska, J., Lopez-Rodriguez, C. *et al.* (2018) Roquin targets mRNAs in a 3'-UTR-specific manner by different modes of regulation. *Nat Commun*, **9**, 3810.
2. Zuker, M. (2003) Mfold web server for nucleic acid folding and hybridization prediction. *Nucleic Acids Res*, **31**, 3406-3415.
3. Gruber, A.R., Lorenz, R., Bernhart, S.H., Neubock, R. and Hofacker, I.L. (2008) The Vienna RNA websuite. *Nucleic Acids Res*, **36**, W70-74.
4. Svergun, D.B., C.; Koch, M. H. J. (1995) CRYSOL - a Program to Evaluate X-ray Solution Scattering of Biological Macromolecules from Atomic Coordinates. *J. App. Cryst.*, **28**, 768-773.
5. Chojnowski, G.Z., R.; Magnus, M.; Bujnicki, J.M. (2021) RNA fragment assembly with experimental restraints. *bioRxiv*.
6. Kent, W.J., Sugnet, C.W., Furey, T.S., Roskin, K.M., Pringle, T.H., Zahler, A.M. and Haussler, D. (2002) The human genome browser at UCSC. *Genome Res*, **12**, 996-1006.
7. Lee, B.T., Barber, G.P., Benet-Pages, A., Casper, J., Clawson, H., Diekhans, M., Fischer, C., Gonzalez, J.N., Hinrichs, A.S., Lee, C.M. *et al.* (2022) The UCSC Genome Browser database: 2022 update. *Nucleic Acids Res*, **50**, D1115-D1122.
8. Blanchette, M., Kent, W.J., Riemer, C., Elnitski, L., Smit, A.F., Roskin, K.M., Baertsch, R., Rosenbloom, K., Clawson, H., Green, E.D. *et al.* (2004) Aligning multiple genomic sequences with the threaded blockset aligner. *Genome Res*, **14**, 708-715.
